# Supplementary material for: Estimating the equity impacts of the smoking ban in England on cotinine levels: a regression discontinuity design
Source: BMJ Open. 2021 Sep 19;11(9):e049547. doi: 10.1136/bmjopen-2021-049547 (PMC8458322; doi:10.1136/bmjopen-2021-049547)
Supplement: Supplementary data [file bmjopen-2021-049547supp001.pdf]

A Appendix

A.1 Sensitivity: Cotinine

Throughout the paper we use log(cotinine +1) as our outcome variable. We use this transformed variable due to the highly right skewed nature of cotinine and the potential impact of extreme values on results. Here we present the main results using cotinine, rather than log cotinine. Throughout results are robust to the choice of variable, but standard errors tend to increase for cotinine.

As in the main analysis, for the average treatment effect there are large and significant reductions for non-smokers, and no significant reductions for smokers.

Table A1: Cotinine: Regression Discontinuity

	(1)<br>**Non-Smokers**<br>Coef./S.E.	(2)<br>**Smokers**<br>Coef./S.E.
Treatment	-4.9852**	-12.1932
	(2.5121)	(32.1071)
Time	0.0016	-0.0230
	(0.0293)	(0.2154)
Treatment X Time	0.0501	-0.0729
	(0.0335)	(0.3449)
**Constant**	5.3445**	301.0218***
	(2.4040)	(18.6132)
N	2916	759
R-squared	0.0026	0.0030

* $p < 0.10$, ** $p < 0.05$, *** $p < 0.01$

For conditional average treatment effects, we observe no significant effects for smokers conditional on any variable. For non-smokers, we observe both significant effects and differences between subgroups. The difference between age groups here disappears and standard errors do increase in comparison to the main results. When we estimate these effects using negative binomial regressions, however, these standard errors reduce.

Figure A1: Forest Plot of CATEs of Cotinine: Non-Smokers and Smokers

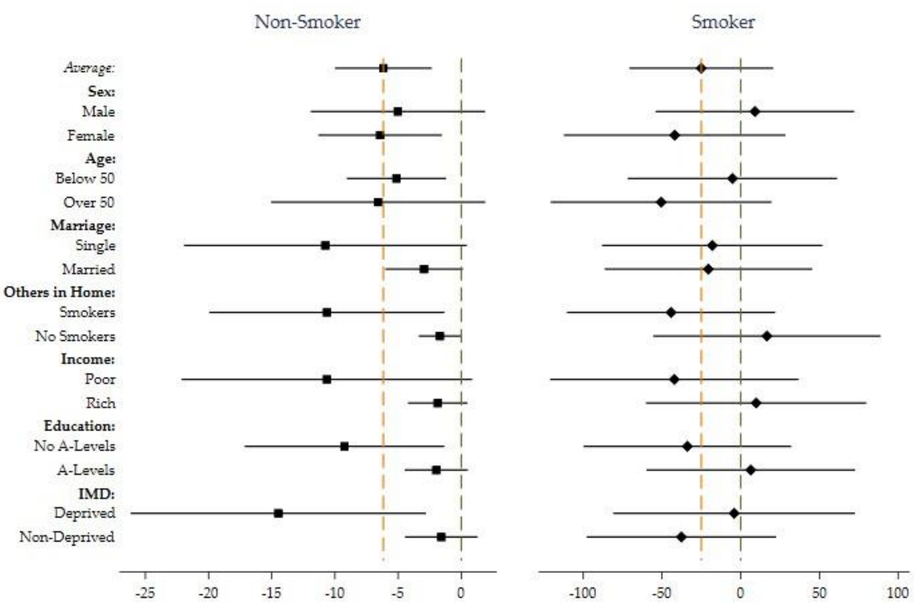

Quantile treatment effects are similar in significance and direction to the main results. There are significant, and increasing, treatment effects for higher quantiles for non-smokers and no significant effects for smokers, at any quantile.

Figure A2: Quantile Treatment Effects: Cotinine

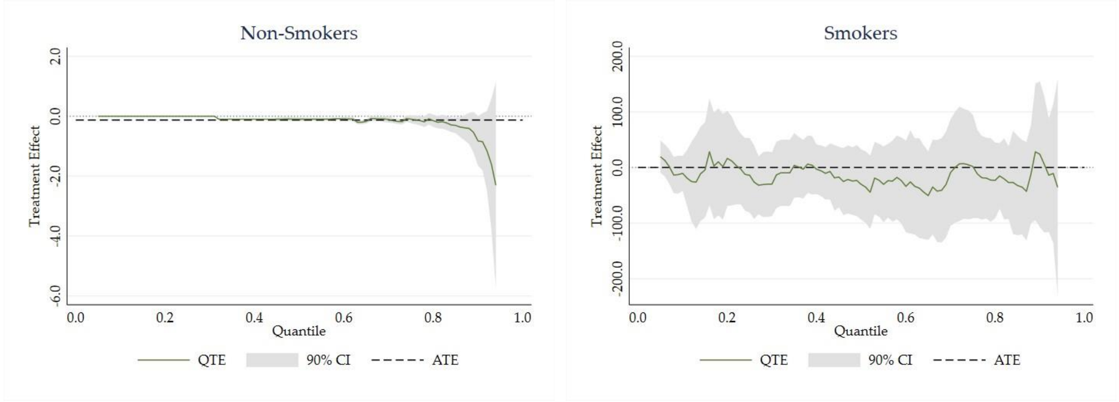

Inequality treatment effects are very similar to the main results. The concentration of cotinine amongst deprived non-smokers was significantly reduced, whilst no significant effects were observed at the sample level or for smokers.

Table A2: Cotinine: Concentration Index

	(1)	(2)	(3)
	**Sample**	**Non-Smokers**	**Smokers**
	Coef./S.E.	Coef./S.E.	Coef./S.E.
Treatment	-0.0209	0.5571***	-0.0728
	(0.1031)	(0.1647)	(0.0648)
Time	0.0011	-0.0062**	0.0006
	(0.0008)	(0.0024)	(0.0004)
Treatment X Time	-0.0018	0.0045	-0.0007
	(0.0012)	(0.0031)	(0.0007)
**Constant**	-0.1335**	-0.5714***	0.0356
	(0.0609)	(0.1835)	(0.0379)
N	3675	2916	759
R-squared	0.0010	0.0026	0.0060

* $p < 0.10$, ** $p < 0.05$, *** $p < 0.01$

A.2 Covariate Balance and Weights

A.2.1 Covariate Balance

Central to the estimation of treatment effects is the comparability of characteristics of the control and treated groups. If there are imbalances in covariates the estimated effects may be biased. To test if there are significant differences in covariates, $x_j, \ \forall j \in J$, the covariate of interest can be regressed against the treatment dummy, *D*:

$$\boldsymbol{x_j} = \beta_0 + \boldsymbol{D}\beta_1 + \boldsymbol{u} \tag{8}$$

Here $\boldsymbol{\beta_1}$ identifies the extent and significance of covariate imbalance between control and treated groups. In regression discontinuity designs, however, the crucial assumption is that there are no significant discontinuities in covariates at the *threshold*. Imbalances between control and treated groups *on average* are not important if this assumption holds. In a simple linear framework, this assumption can be tested by regressing the covariates against the treatment dummy, *D*, (centred) forcing variable, *Z* and their interaction:

$$\boldsymbol{x}_j = \gamma_0 + \boldsymbol{D}\gamma_1 + \boldsymbol{Z}\gamma_2 + (\boldsymbol{D}_0 \times \boldsymbol{Z})\gamma_3 + \boldsymbol{u}, \qquad for\ |\boldsymbol{Z}| \leq h \tag{9}$$

If $\boldsymbol{\gamma_1}$ is significant it shows there is a discontinuity in $\boldsymbol{x}_j$ at the threshold. This imbalance at the threshold could bias the estimation of the treatment effect.

A.2.2 Inverse Probability of Treatment Weights

To mitigate any potential biases that these imbalances could cause, doubly-robust methods which use propensity weights alongside the regression discontinuity design, can be used. We estimate the inverse probability of treatment to use as weights. This balances covariates across treatment groups, giving lower weight to those characteristics which are predictors of treatment (i.e. via selection into treatment).

To estimate propensity weights the following regression is run, as a logit:

$$\boldsymbol{D} = \delta_0 + \sum_{j}^{J} \left(\boldsymbol{X_j} \delta_{1j} + \left(\boldsymbol{X_j'} \times |\boldsymbol{Z}| \right) \delta_{2j} \right) + \boldsymbol{u} \qquad for\ |\boldsymbol{Z}| \leq h \tag{10}$$

This formulation includes both covariates $\boldsymbol{X_j}$ and the interaction between those covariates and the absolute value of the forcing variable **Z**. This particular formulation is used to ensure balance not just in expectation (as in Equation (8)) but also at the discontinuity. By using the absolute value of **Z** we provide this flexibility, whilst ensuring the overlap assumption is not violated. These weights are estimated separately for each subgroup.

Using this we predict the probability of being treated, given the observable characteristics $Pr(D = 1|X, Z)$. The inverse probability of treatment weights are then calculated as:

$$w_i^{IPT} = \frac{D}{\Pr(D = 1\ |X, Z)} + \frac{1 - D}{1 - \Pr(D = 1\ |X, Z)} \tag{11}$$

Table A3 shows the consequence of using these weights on covariate balance. Each coefficient (and standard error) are results from separate regressions, with the outcome variable x_j in the first column. Average differences are the β_1 coefficients of Eq.(8), with differences at the discontinuity being the γ_1 coefficients of Eq.(9).

In the unweighted sample we see that the treated group, on average, live in more rural and less deprived areas. At the discontinuity, respondents have significantly fewer children, are more likely to be retired and live in rural areas. These differences could potentially cause bias in the estimates of the treatment effect and need to be controlled for. When the weights are applied, however, we observe no significant differences either on average or at the discontinuity; removing the selection bias (on observables) which could have biased estimates.

Table A3: Balance

	Unweighted		Weighted	
	(1)	(2)	(3)	(4)
	**Average**	**Discontinuity**	**Average**	**Discontinuity**
	Coef./Std. err.	Coef./Std. err.	Coef./Std. err.	Coef./Std. err.
Age	0.6179	3.5169***	-0.0543	-0.2464
	(0.5288)	(1.0511)	(0.6107)	(1.1586)
Sex (1 = female)	0.0025	-0.0051	0.0005	0.0019
	(0.0149)	(0.0297)	(0.0175)	(0.0337)
Married Couple	-0.0155	-0.0023	-0.0004	0.0026
	(0.0148)	(0.0296)	(0.0173)	(0.0331)
Number of Children	-0.0248	-0.2123***	-0.0007	0.0145
	(0.0239)	(0.0445)	(0.0298)	(0.0592)
Retired	0.0153	0.0829***	-0.0016	-0.0038
	(0.0132)	(0.0266)	(0.0149)	(0.0283)
Ethnicity (1 = white)	0.0170**	0.0089	-0.0023	-0.0056
	(0.0085)	(0.0166)	(0.0106)	(0.0207)
Urban	-0.0088	-0.1027***	0.0034	0.0069
	(0.0126)	(0.0249)	(0.0141)	(0.0254)
IMD Quintile (1 = deprived)	0.0880**	0.1039	-0.0013	-0.0064
	(0.0418)	(0.0838)	(0.0496)	(0.0960)
Occupation: Manager	0.0065	0.0202	-0.0017	-0.0085
	(0.0142)	(0.0284)	(0.0165)	(0.0316)
Education: A-Level	0.0183	-0.0047	-0.0023	-0.0040
	(0.0149)	(0.0297)	(0.0175)	(0.0336)
Home Owner	0.0419***	-0.0038	0.0024	0.0043
	(0.0130)	(0.0259)	(0.0152)	(0.0289)

* $p < 0.10$, ** $p < 0.05$, *** $p < 0.01$

A.2.3 Triangular Kernels

We run each regression discontinuity design as a locally-weighed regression, using triangular kernels to give higher weights to observations closer to the threshold. These weights are calculated as:

$$w_i^{TRI} = h - |Z_i|$$

The highest weights are at the threshold ($Z = 0$) and decrease linearly as the bound of the bandwidth, h is reached. These are multiplied by the inverse probability of treatment weights to provide the final weights ($w_i = w_i^{IPT} . w_i^{TRI}$) for the weighted regressions run:

$$\sqrt{w_i}\,\boldsymbol{y} = \sqrt{w_i}\,(\beta_0 + \boldsymbol{D}\beta_1 + \boldsymbol{Z}\beta_2 + (\boldsymbol{D} \times \boldsymbol{Z})\beta_3 + \boldsymbol{u}) \qquad for\ |\boldsymbol{Z}| \leq h \tag{12}$$

A.3 Baseline Characteristics

Table A4 summarises the baseline characteristics of the control group, by cotinine quintile. Smoking variables are strongly associated with cotinine, with higher numbers of smokers, higher intensity of smoking and higher exposure to smoke at higher cotinine quintiles. Those with lower levels of cotinine are older, more likely to be female, married and retired, to live in rural areas and have more children. Higher socioeconomic status individuals have lower levels of cotinine, for a range of variables: IMD, income, occupation, education and home ownership. Additionally, those with higher cotinine levels have lower self-assessed health, more limiting longstanding illnesses and they drink more heavily.

Table A4: Descriptive Statistics by Cotinine Quintile

	**Cotinine Quintile**					**Summary**
	**Q1**	**Q2**	**Q3**	**Q4**	**Q5**	**Mean**
	Mean	Mean	Mean	Mean	Mean	Mean
**Cotinine Levels**						
Cotinine	0.00	0.14	0.38	7.61	338.18	69.13
Log Cotinine	0.00	0.13	0.32	1.37	5.68	1.47
**Smoking**						
Smoker	0.01	0.00	0.00	0.12	0.93	0.21
Packs Smoked Per Day	0.00	0.00	0.00	0.14	2.40	0.51
Exposed to Smoke	0.35	0.33	0.44	0.60	0.72	0.48
Hours Exposed to Smoke	1.22	1.18	2.95	7.65	12.37	4.89
Ever Smoked	0.45	0.49	0.54	0.61	0.99	0.61
Smoke in Pubs	0.00	0.00	0.00	0.06	0.28	0.07
Exposed in Pubs	0.16	0.16	0.25	0.38	0.38	0.26
**Demographics**						
Age	52.18	52.35	50.11	48.23	44.80	49.70
Sex (1 = female)	0.54	0.56	0.54	0.52	0.50	0.53
Married Couple	0.67	0.65	0.65	0.54	0.42	0.59
Number of Children	0.46	0.37	0.47	0.40	0.54	0.44
Retired	0.29	0.31	0.28	0.25	0.11	0.25
Ethnicity (1 = white)	0.90	0.91	0.89	0.90	0.92	0.91
Urban	0.76	0.72	0.71	0.80	0.84	0.77
**Socioeconomic Status**						
IMD Quintile (1 = deprived)	3.47	3.44	3.25	2.94	2.62	3.16
Log Equivalised Income	10.14	10.19	10.17	10.01	9.79	10.06
Occupation: Manager	0.42	0.41	0.38	0.31	0.22	0.35
Education: A-Level	0.50	0.49	0.49	0.44	0.33	0.45
Home Owner	0.84	0.78	0.78	0.73	0.54	0.74
**Health Variables**						
Self Assessed Health (5 = v. good)	4.09	4.18	4.08	3.89	3.68	3.99
Limiting Longstanding Illness	0.27	0.23	0.25	0.26	0.33	0.27
Alcohol Units: Heaviest Day	5.34	5.42	6.88	7.81	9.49	6.89

A.4 Average Treatment Effects

Table A5 shows the results from the regression discontinuity design. The average treatment effect of the ban on non-smokers is large and significant, at -0.128, whilst there is no significant effect for smokers. The constant shows the expected level of log cotinine exposure at the time of the ban, for the control group: 0.399 for non-smokers and 5.343 for smokers.

Table A5: Log Cotinine: Regression Discontinuity

	(1)	(2)
	**Non-Smokers**	**Smokers**
	Coef./S.E.	Coef./S.E.
Treatment	-0.1279**	0.0385
	(0.0632)	(0.1662)
Time	-0.0004	-0.0001
	(0.0005)	(0.0012)
Treatment X Time	0.0010	-0.0006
	(0.0008)	(0.0018)
**Constant**	0.3987***	5.3434***
	(0.0395)	(0.1089)
N	2952	766
R-Squared	0.0062	0.005

$^{*}p < 0.10$, $^{**}p < 0.05$, $^{***}p < 0.01$

Figure A3 shows the regression discontinuity plot for smokers and non-smokers. This shows the (weighted) mean log cotinine (averaged across two days) plotted against the number of days from the ban. The line plot shows the predicted point estimates from the regression of Table A5, whilst the area plot shows the 90% confidence intervals. We observe a discontinuity at the time of the ban for non-smokers, but not smokers. These are the average treatment effects observed in Table A5.

This figure is indicative of the quantile treatment effects we observe in Figure 3: many observations at the lower values of log cotinine move the small distance towards zero, whilst those higher values of log cotinine become much less frequent following the ban.

Figure A3: Regression Discontinuity Plot: Non-Smokers and Smokers

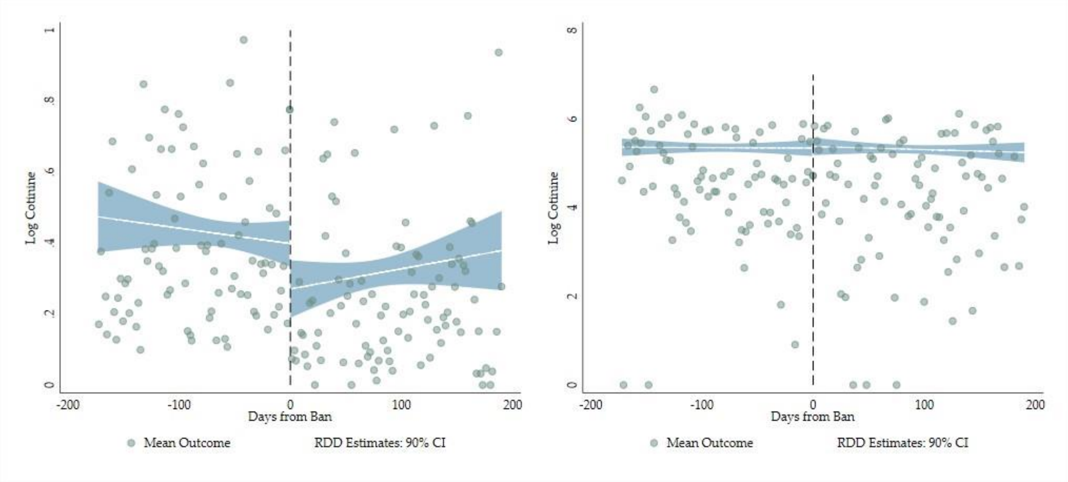

A.5 Sensitivity Analysis

Figure A4 shows a forest plot of sensitivity analysis for the estimated average treatment effects. The baseline point estimate of Table 3 is shown at the top, with 90% confidence intervals, for non-smokers and smokers. The estimates below show how sensitive these results are to changes in the functional form of the regression discontinuity, the size of the bandwidth, the weights used and to the addition of controls.

Figure A4: Sensitivity Forest Plot: Log Cotinine, Non-Smokers and Smokers

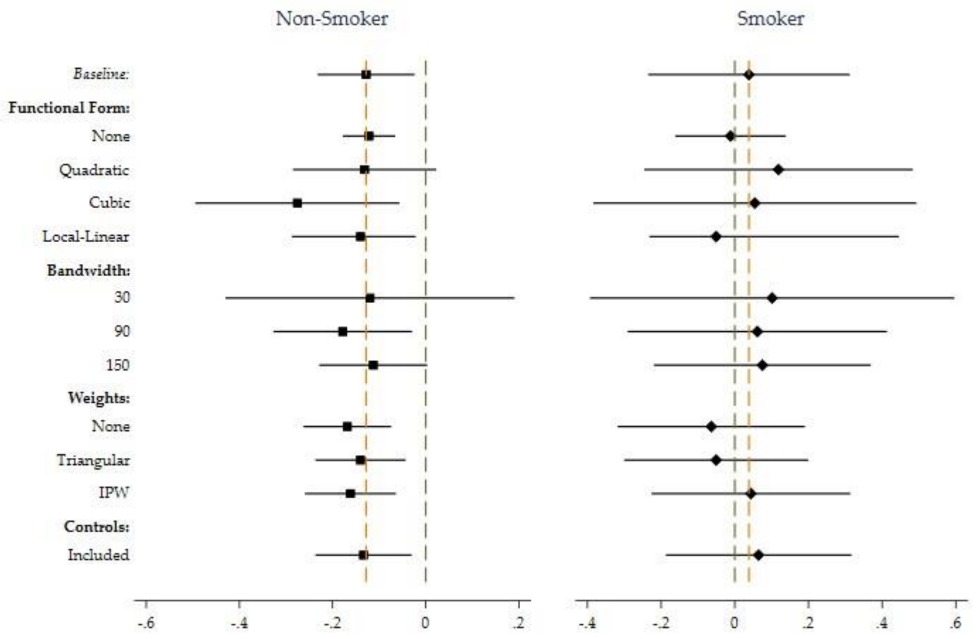

Results show that the analysis appears robust to each of these sensitivity checks. For non-smokers, the effects are never significantly different from the baseline results (dotted orange line) and (with exception to the use of a small bandwidth and quadratic functional form) the estimated effects are always significant, at the 90% level. For smokers, the effects are never significantly different from the baseline effect nor are they significantly different from zero, and indeed appear to hover around a zero effect.

A.6 Predicted Conditional Potential Outcomes

Figure A5 plots the predicted potential outcomes conditional on observable characteristics. This shows the expected levels of log cotinine at the threshold, for control (red) and treated (blue) groups. These results should be used to supplement the estimated treatment effects in Figure 2; the treatment effects are the differences between these potential outcomes.

Figure A5: Forest Plot of Potential Outcomes of Log Cotinine: Non-Smokers and Smokers

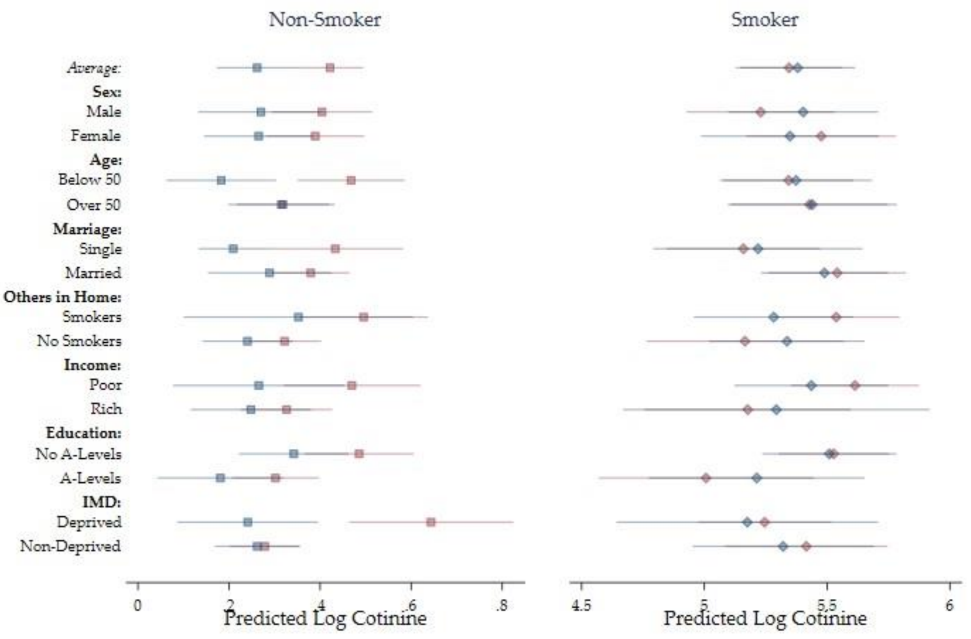

Non-smokers are found to have both significant differences between control and treated potential outcomes (i.e. significant treatment effects) and are found to have differences in exposure levels between subgroups. After the ban the differences between those subgroups that are different before, is much reduced. For smokers, there are no significant differences between potential outcomes; neither between control and treated, nor between subgroups.
