# Supplementary material for: Estimating the equity impacts of the smoking ban in England on cotinine levels: a regression discontinuity design
Source: BMJ Open. 2021 Sep 19;11(9):e049547. doi: 10.1136/bmjopen-2021-049547 (PMC8458322; doi:10.1136/bmjopen-2021-049547)
Supplement: Author's manuscript [file bmjopen-2021-049547.draft_revisions.pdf]

BMJ Open is committed to open peer review. As part of this commitment we make the peer review history of every article we publish publicly available.

When an article is published we post the peer reviewers' comments and the authors' responses online. We also post the versions of the paper that were used during peer review. These are the versions that the peer review comments apply to.

The versions of the paper that follow are the versions that were submitted during the peer review process. They are not the versions of record or the final published versions. They should not be cited or distributed as the published version of this manuscript.

BMJ Open is an open access journal and the full, final, typeset and author-corrected version of record of the manuscript is available on our site with no access controls, subscription charges or pay-per-view fees (http://bmjopen.bmj.com).

If you have any questions on BMJ Open's open peer review process please email info.bmjopen@bmj.com

BMJ Open

Equity Impacts of the Smoking Ban in England

Journal:	*BMJ Open*
Manuscript ID	bmjopen-2021-049547
Article Type:	Original research
Date Submitted by the Author:	28-Jan-2021

Complete List of Authors:	Robson, Matthew; University of York Department of Health Sciences, Department of Health Sciences<br>Lord, Joseph; University of York Centre for Health Economics, Centre for Health Economics<br>Doran, Tim; University of York Department of Health Sciences, Department of Health Sciences

Keywords:	HEALTH ECONOMICS, PUBLIC HEALTH, STATISTICS & RESEARCH METHODS

SCHOLARONE™
Manuscripts

I, the Submitting Author has the right to grant and does grant on behalf of all authors of the Work (as defined in the below author licence), an exclusive licence and/or a non-exclusive licence for contributions from authors who are: i) UK Crown employees; ii) where BMJ has agreed a CC-BY licence shall apply, and/or iii) in accordance with the terms applicable for US Federal Government officers or employees acting as part of their official duties; on a worldwide, perpetual, irrevocable, royalty-free basis to BMJ Publishing Group Ltd ("BMJ") its licensees and where the relevant Journal is co-owned by BMJ to the co-owners of the Journal, to publish the Work in this journal and any other BMJ products and to exploit all rights, as set out in our licence.

The Submitting Author accepts and understands that any supply made under these terms is made by BMJ to the Submitting Author unless you are acting as an employee on behalf of your employer or a postgraduate student of an affiliated institution which is paying any applicable article publishing charge ("APC") for Open Access articles. Where the Submitting Author wishes to make the Work available on an Open Access basis (and intends to pay the relevant APC), the terms of reuse of such Open Access shall be governed by a Creative Commons licence – details of these licences and which Creative Commons licence will apply to this Work are set out in our licence referred to above.

Other than as permitted in any relevant BMJ Author's Self Archiving Policies, I confirm this Work has not been accepted for publication elsewhere, is not being considered for publication elsewhere and does not duplicate material already published. I confirm all authors consent to publication of this Work and authorise the granting of this licence.

For peer review only

Equity Impacts of the Smoking Ban in England

Matthew Robson[1], Joseph Lord[2] & Tim Doran[3]

January 26th, 2021

Abstract

Objective. To estimate the equity impacts of the 2007 smoking ban in England, for both smokers and non-smokers.

Design. Doubly-robust regression discontinuity analysis of salivary cotinine levels. Conditional average treatment effects were used to estimate differential impacts of the ban by socioeconomic deprivation (based on the Index of Multiple Deprivation). Distributional impacts were further assessed using conditional quantile treatment effects and inequality treatment effects.

Setting. In 2007, England introduced a ban on smoking in public places. This had little impact on tobacco consumption by smokers, but was associated with decreases in environmental tobacco smoke exposure for non-smokers. However, the impact of the ban on socioeconomic inequalities in exposure are unclear.

Participants. 766 smokers and 2,952 non-smokers responding to the Health Survey for England in 2007.

Outcome Measure. Levels of salivary cotinine.

Results. Before the ban, socioeconomic deprivation was associated with higher cotinine levels for non-smokers, but not for smokers. The ban caused a significant reduction in average cotinine levels for non-smokers (p=0.043), but had no effect for smokers (p=0.817). Reductions for non-smokers were greater for more deprived groups with higher levels of exposure, and there was a significant reduction in socioeconomic-related inequality in cotinine. Across the whole population (both smokers and non-smokers), there was no significant increase in the concentration of cotinine levels amongst the socioeconomically deprived.

Conclusion. The 2007 ban on smoking in public places had little impact on smokers, but was, as intended, associated with reductions in both i) average levels of environmental tobacco smoke exposure, and ii) deprivation-related inequality in exposure among non-smokers.

Keywords: Health Economics, Public Health, Statistics and Research Methods.

Wordcount: 3080

[1] Department of Health Sciences, University of York, York, United Kingdom. Email: matthew.robson@york.ac.uk.
[2] Centre for Health Economics, University of York, York, United Kingdom. Email: josephlord534@gmail.com.
[3] Corresponding author: Tim Doran, Address: Department of Health Sciences, University of York, York, North Yorkshire, GB. Email: tim.doran@york.ac.uk. Website: https://equipol.org. Phone: 01904 321524.

For peer review only

Strengths and limitations of this study

- We used a doubly-robust regression discontinuity design to derive causal estimates of the equity impacts of the smoking ban for both smokers and non-smokers.
- Data were derived from a rich, individual-level dataset with an objective measure of tobacco smoke exposure to avoid social desirability bias in reported variables.
- Causal effects of the ban were estimated across the entire distribution of the outcome, identifying conditional distributional effects and the impact on socioeconomic-related inequality.
- We focused only upon the short-term effects of the ban and rely on an area-based measure of socioeconomic status.

For peer review only

INTRODUCTION

Successive UK governments have pursued a range of public health policies to reduce population harms from tobacco smoke exposure since links to lung cancer and other common fatal conditions were confirmed in the 1950s[1]. With public education, increased taxation and restrictions on sales and advertising, smoking prevalence fell from 70% in the 1950s to under 25% percent by the turn of the century[2]. More coercive policies began to be considered in the 1990s as studies emerged identifying harmful impacts on non-smokers exposed to environmental smoke[3-12], including exposure at home[7,8] and in the workplace[13]. Estimates of the population impact of environmental exposure suggest that over 10 percent of smoking-related deaths may occur in non-smokers[14], and the risks to smokers themselves are further increased by environmental smoke. The impacts of smoking are also unevenly distributed; whilst rates of smoking have fallen in all socioeconomic groups since the 1950s, they fell fastest in the most affluent groups. At the end of the century, over half of the difference in mortality between men in high (professional, managerial and technical) and low (unskilled manual) socioeconomic groups was attributable to tobacco exposure[15].

The growing weight of evidence on environmental tobacco smoke eventually led to the implementation of national bans in Scotland (March 2006), Wales and Northern Ireland (April 2007), and England (July 2007). For England and Wales, the Health Act 2006 effectively prohibited tobacco smoking in enclosed public places, including workplaces, public transport and private clubs. The ban had little impact on tobacco consumption[16-19], but smokers smoked substantially less in enclosed public places, including at work and in pubs[17]. Evidence on the equity impacts of the smoking ban, particularly for non-smokers, is less clear. In England, smoking prevalence continued to fall at similar rates across different socioeconomic status (SES) groups after the ban, preserving the gaps between them; by 2015 smoking prevalence remained above 25% in the most deprived fourth and fifth of areas, compared with 10% in the least deprived fifth[19].

In terms of second-hand exposure, Semple et al. noted that pubs in more deprived areas, with higher baseline particulate (PM2.5) concentrations, tended to have greater reductions after the ban[20]. In contrast, King et al. found that higher SES smokers were more likely than low status smokers to prohibit smoking in their own home following the ban[21].

For peer review only

Obtaining more direct evidence on the equity impacts of smoking bans on non-smokers is subject to two key limitations: a reliance on before-and-after study designs measuring association, rather than strong econometric designs identifying causal effects; and a focus on average effects for the whole population, rather than distributional effects relating to baseline exposure to tobacco smoke and socioeconomic circumstances. The latter limitation is particularly important, as national policies of this kind are frequently less effective for less affluent socioeconomic groups with higher baseline levels of exposure, and this has the unintended consequence of widening existing health inequalities. In this paper, we assess the impact of the national smoking ban on cotinine levels, a biomarker of tobacco smoke exposure, for both smokers and non-smokers. We use approaches that assess the full range of effects in relation to socioeconomic characteristics across the entire distribution of exposure levels, to more accurately determine the equity impacts of the smoking ban.

METHODS

Intervention

The national smoking ban in England was implemented on 1st July 2007. Smoking in enclosed public places became an offence, subject to fines of up to £200 for individuals and £2,500 for businesses. Exemptions were included, at that time, for psychiatric units, nursing homes, prisons and other specified public places.

Data

Data were derived from the Health Survey for England (HSE), an annual cross-sectional survey which records health and lifestyle changes over time in England at the individual and household level. The dataset includes variables covering tobacco smoking and exposure, as well as a range of individual characteristics recorded by an interview and, if consent is given, a follow-up nurse's visit (on average 18.4 days later). Our main analysis uses data from the 2007 wave of the survey, covering the period of the introduction of the public smoking ban in England.

For peer review only

Patient and Public Involvement

As we conduct a secondary analysis of survey data, there was no direct patient and public involvement.

Tobacco Exposure

The primary outcome is exposure to tobacco smoke, estimated by salivary cotinine concentration. Cotinine is a metabolite of nicotine which diffuses from the bloodstream into saliva, making salivary cotinine concentration a convenient and reliable biomarker of regular tobacco exposure[22]. Salivary cotinine concentrations above 12ng/ml indicate smoking status with high sensitivity and specificity[23] and scale with the extent of tobacco use. Lower concentrations are also detectable, so cotinine is useful for monitoring changes in both tobacco use by smokers and exposure to second-hand smoke in non-smokers. Distributions of salivary cotinine levels tend to be heavily skewed; for non-smokers, levels are often undetectable whereas for smokers a minority of concentrations are extremely high, and these can have a disproportionate impact on results. We therefore used log(cotinine+1) as our primary outcome, but also report results using untransformed values in Appendix A.1. The subset of respondents to the HSE receiving a nurse visit are asked to provide a saliva sample, which is then analysed for cotinine concentration. For 2007, 4,058 valid cotinine measurements were available. Current smoking status (smoker/non-smoker) was based on self-report.[1]

Socioeconomic deprivation

Our primary measure of socioeconomic deprivation was the 2007 Index of Multiple Deprivation (IMD) quintile[24]. This is an area-based measure linked to postcode, for which we use the quintiles or group into "deprived" (the lowest two quintiles) and "non-deprived" (highest three quintiles). IMD was selected over alternative SES variables, such as household income and occupation, as it had fewer

[1] In the pre-intervention sample, 95.7% of self-reported smokers and 4.3% of non-smokers had cotinine concentrations >12ng/ml.

For peer review only

missing data, demonstrated the largest differential conditional effect, and was more stable during the period between the main survey and nurse follow-up.

Analysis

Regression Discontinuity Design

We used a sharp regression discontinuity (RD) design to estimate the causal effects of the intervention. The design uses 1st July 2007, the date of implementation of the national smoking ban, as a threshold dividing the sample into 'control' (interviewed before the ban) and 'treated' (interviewed after the ban): with a bandwidth of +/- 190 days. Doubly-robust methods were used, with inverse probability of treatment weights in locally-weighted regressions with triangular kernels, to control for potential covariate imbalances at the time of the ban. More details are provided in Appendix A.2.

The model takes the general form:

$$\boldsymbol{y} = \beta_0 + \boldsymbol{D}\beta_1 + \boldsymbol{Z}\beta_2 + (\boldsymbol{D} \times \boldsymbol{Z})\beta_3 + \boldsymbol{u} \quad for\ |\boldsymbol{Z}| \leq h \#(1)$$

Where **y** is the outcome variable (log cotinine), **D** is the treatment dummy and **Z** is the forcing variable, time from ban, which takes the value zero on the day of the policy change, is positive if the observation is after the ban, and negative before. **u** is the error term, which is assumed to be independent and identically distributed with an expected value of zero. For the coefficients, β_0 represents the average outcome for the control group at the point of discontinuity where $Z \to 0$ (date of the ban), β_1 is the treatment effect at the discontinuity, β_2 is the time trend before the ban, and β_3 indicates the change in the trend after the ban.

Treatment Effects

We used the RD approach to estimate the potential outcomes individuals would have had with and without treatment at the threshold[25]. The overall impact of the smoking ban on tobacco exposure was measured as an average treatment effect:

For peer review only

$$ATE = \beta_1 = E[\boldsymbol{Y}(1) - \boldsymbol{Y}(0) \,|\, \boldsymbol{Z} = 0] \#(2)$$

Where Y(0) is the *potential* outcome an individual would have had without treatment and Y(1) is the *potential* outcome with treatment. We then evaluated the distributional impacts of the smoking ban using a series of approaches: i) Conditional Average Treatment Effects ii) Quantile Treatment Effects; iii) Conditional Quantile Treatment Effects; and iv) Inequality Treatment Effects.

Conditional Average Treatment Effects (CATEs) estimate the average effect of the treatment (the smoking ban) on y (log cotinine) conditional on x (e.g. whether the effect of the smoking ban varied with socioeconomic deprivation):

$$CATE(\tilde{\boldsymbol{x}}) = E[\boldsymbol{Y}(1) - \boldsymbol{Y}(0) \,|\, \tilde{\boldsymbol{X}} = \tilde{\boldsymbol{x}}, \boldsymbol{Z} = 0] \#(3)$$

Quantile Treatment Effects estimate the effect of the treatment on *y* at different quantiles (τ) of *y*:

$$QTE(\tau) = Q^{\tau}_{Y(1)|\boldsymbol{Z}=0} - Q^{\tau}_{Y(0)|\boldsymbol{Z}=0} \#(4)$$

Where $Q^{\tau}_{Y(.)|\boldsymbol{Z}=0}$ is the quantile function which returns the cotinine level Y(.) at the threshold for each quantile, $\tau \in (0,1)$ (conditional rank of the outcome). The differences between these quantile functions gives the treatment effect at each quantile. These effects are estimated directly, using quantile regressions[26] within the RD framework[27]. This allows us to determine how the ban changed the distribution of tobacco exposure, revealing whether levels fell more at the top or bottom of the distribution.

Conditional Quantile Treatment Effects (CQTEs) combine QTEs and CATEs, estimating the effect of the treatment at the quantile of y (log cotinine), conditional on x (e.g. socioeconomic deprivation):

$$CQTE(\tau, \tilde{\boldsymbol{x}}) = Q^{\tau}_{Y(1)|\tilde{\boldsymbol{X}} = \tilde{\boldsymbol{x}},\ \boldsymbol{Z}=0} - Q^{\tau}_{Y(0)|\tilde{\boldsymbol{X}} = \tilde{\boldsymbol{x}},\ \boldsymbol{Z}=0} \#(5)$$

Finally, Inequality Treatment Effect (ITEs)[28] identify the difference in inequality indices between the treated and control:

$$ITE = W(Y(1) \mid \boldsymbol{Z} = 0) - W(Y(0) \mid \boldsymbol{Z} = 0)\#(6)$$

Where $W(.)$ denotes an inequality index, in this case the Concentration Index[29], a *relative* measure of bivariate inequality:

$$W(y,\tilde{x}) = \frac{1}{N}\sum_{i=1}^{N}\left((2R(\tilde{x}_i) - 1)\frac{y_i}{\bar{y}}\right) \#(7)$$

Where $(2R(\tilde{x}_i) - 1)$, is the fractional rank of $\tilde{x}_i$, and $\bar{y} = \frac{1}{N}\sum_i^N(y_i)$, the mean outcome. The concentration index indicates how concentrated health-related outcomes are amongst those of a higher/lower socioeconomic status. Where there is no socioeconomic-related inequality, the concentration index is zero, with values further from zero indicating greater inequality. We used methods developed by Heckley et al. to estimate ITEs within the regression discontinuity framework using recentered influence function regressions[30].

RESULTS

Baseline Characteristics

Figure 1 shows the distribution of transformed cotinine values (log(cotinine+1)) for non-smokers and smokers in the control group (pre-intervention). For non-smokers, the distribution was heavily right skewed (31.4% of the sample had zero values) with a mean of 0.43 (actual value 4.95 ng/ml). For smokers, the distribution was left skewed with a mean of 5.37 (303.24 ng/ml).

[INSERT FIGURE 1]

Baseline characteristics, by quintile of cotinine, are shown in Table 5, in Appendix A.3. Higher cotinine levels were associated with younger age, being unmarried, living in an urban environment,

For peer review only

lower socioeconomic status, poorer self-assessed health and higher levels of alcohol consumption, in addition to smoking status and tobacco consumption.

Average Treatment Effects

At the time of the ban, the expected baseline level of log cotinine (without treatment) was 0.40 for non-smokers and 5.34 for smokers. There was a large and significant reduction in cotinine concentrations for non-smokers (-0.128, p=0.043), but no significant effect of the ban for smokers (0.036, p=0.817). The regression outputs are shown in Appendix A.4. These results are robust to a set of sensitivity analyses (see Appendix A.5). These effects are shown at the top of Figure 2, which shows forest plots of average effects of the smoking ban conditional on sex, age, marital status, smoking status of others in the household, equalised household income, highest qualification and IMD (CATEs). For smokers there were no significant effects conditional on any variable. For non-smokers, there were both significant effects and significant differences between subgroups, most notably larger reductions in cotinine levels for younger, single, income poor or IMD deprived respondents.

The differences in CATEs for non-smokers are partially explained by different baseline predictions across subgroups. For example, the predicted average level of log cotinine at the time of the intervention was 0.65 for deprived and 0.30 for non-deprived. After the ban these levels fell to 0.23 and 0.27 respectively (not significantly different), see Figure 6 in Appendix A.6 for more detail.

[INSERT FIGURE 2]

Distributional Analysis

Figure 3 shows Quantile Treatment Effects (QTEs), i.e. the effect of the smoking ban on the level of cotinine at different parts of the distribution (quantiles of cotinine). For non-smokers, there was no effect on cotinine levels at the lower quantiles (which were already at 0) but significant, and increasing, reductions appeared at higher quantiles of cotinine. For smokers, there were no significant effects at any quantile.

[INSERT FIGURE 3]

For peer review only

Figure 4 shows QTEs conditional on IMD deprivation. Significant reductions in cotinine levels are apparent for deprived non-smokers at higher levels of exposure. For non-deprived non-smokers there are significant small effects at the mid-range of exposure (going from very little to zero exposure) but not at higher levels. For both groups of smokers there was no significant reduction at any quantile of cotinine.

[INSERT FIGURE 4]

Changes in Inequality

Table 1 shows the causal effect of the ban on bivariate inequality (the concentration index) i.e. how the concentration of log cotinine levels amongst the socioeconomically deprived (IMD quintile) changes.

The negative constants in models (1) and (2) show that higher levels of cotinine were concentrated amongst more socio-economically deprived respondents at baseline for the whole sample (indicating higher levels of smoking) and non-smokers (indicating higher levels of second-hand exposure). However, the constant is close to zero for smokers, therefore conditional on being a smoker, deprivation has limited impact on cotinine levels.

For peer review only

Table 1: Log Cotinine: Concentration Index

	(1) **Sample** Coef./S.E.	(2) **Non-Smokers** Coef./S.E.	(3) **Smokers** Coef./S.E.
Treatment	0.0265	0.2097**	-0.0285
	(0.0693)	(0.0910)	(0.0182)
Time	0.0005	-0.0015*	0.0003**
	(0.0005)	(0.0008)	(0.0001)
Treatment X Time	-0.0009	0.0007	-0.0003
	(0.0008)	(0.0011)	(0.0002)
**Constant**	-0.1501***	-0.2329***	0.0119

	(0.0394)	(0.0559)	(0.0127)
N	3718	2952	766
R-squared	0.0009	0.0029	0.0076

$^{*}p < 0.10$, $^{**}p < 0.05$, $^{***}p < 0.01$

The treatment effect shows that the ban caused a significant increase in the concentration index for non-smokers (i.e. towards zero), reducing the concentration of cotinine levels amongst deprived non-smokers. No significant effects were found for the whole sample or for smokers. These results indicate that the ban caused a reduction in deprivation related inequality in cotinine levels among non-smokers but had no significant effect for smokers.

DISCUSSION

Given the socioeconomic gradient of tobacco smoke exposure, public smoking bans could potentially benefit deprived groups more. Conversely, poorer individuals may respond less well to such policies due to lack of opportunities and resources, contextual factors[31], and attitudes towards anti-smoking policies[32]. We found the smoking ban in England significantly reduced exposure for non-smokers with greater reductions for socioeconomically deprived groups with higher baseline levels of exposure. However, the ban had no significant impact on exposure levels, or on inequalities in exposure, for smokers.

Strengths and limitations of the study

We used a doubly-robust regression discontinuity design to derive causal estimates of the differential impacts of a national smoking ban across the entire distribution of exposure levels, for both smokers and non-smokers. We also used a rich, individual-level dataset with an objective measure of tobacco smoke exposure to avoid social desirability bias in reported variables and explored differential effects of the ban both between groups using conditional average treatment effects and across the entire distribution of cotinine levels using quantile treatment effects. We unified these approaches to examine conditional distributional effects and estimate the causal impact of the ban on socioeconomic-related inequality in cotinine.

For peer review only

The study had several limitations. First, the HSE is a repeated cross-sectional survey and we were therefore unable to observe individual's over time; rather, we compared distributions of exposure for different individuals surveyed before and after the ban. Second, we relied on self-report to determine smoking status at the time of the survey. The distributions of cotinine levels for smokers and non-smokers suggest that self-report was accurate for the majority of subjects, but there may have been some misclassification. Third, social deprivation was measured by area deprivation rather than by individual social status and was arbitrarily categorised in quintiles, our analysis of socioeconomic deprivation was therefore less fine-grained than our analysis of cotinine levels. Finally, we have only measured the short-term effects of the ban, and these may change over the longer term.

Findings

Our results suggest that the smoking ban in England did little to reduce overall risk or socioeconomic inequalities in smoking related diseases for the smoking population, but it did, as intended, reduce both overall risk and inequalities in risk for non-smokers, with greater reductions in environmental tobacco smoke exposure for socioeconomically deprived non-smokers with higher baseline levels of exposure. These findings align with systematic reviews showing that compulsory smoking restrictions are generally more equitable than voluntary policies[33] and Semple et al.'s findings of greater reductions in environmental smoke in pubs in more deprived areas following the UK ban[20]. A previous study of cotinine levels in non-smokers by Sims et al. similarly reported significant reductions in average exposure following the ban but, in contrast to our results, found no significant reductions in the lowest SES households[34]. Whereas our study used a regression discontinuity approach, Sims et al. modelled impacts based on pre-intervention trends that indicated exposure was already falling prior to 2007. However, these models lacked data for the three years immediately prior to 2007, and may have underestimated additional reductions resulting from the ban. Sims et al. also measured SES using social class of the household head, resulting in a relatively small sample of low status non-smokers; our different results might therefore be explained by reduced levels of exposure being achieved in more deprived areas generally, but not in the very lowest status households. This is consistent with our previous study, which found significant reductions in self-reported exposure for

For peer review only

non-smokers which increased with social deprivation, with the exception of individuals with extremely low SES, for whom there was no significant impact[35].

A major concern at the time of the ban was that smokers would substitute smoking in private areas for smoking in public places, and that this could unintentionally increase risk for vulnerable groups, as happened in the US following similar bans[36]. Our results suggest that such substitution activity did not occur and that there was a net reduction in exposure for adult non-smokers; this is supported by findings of reductions in smoking prevalence inside cars (from 32% to 26%) and the home (from 65% to 55%) reported by smokers following the ban[17].

Conclusions

The 2007 smoking ban in England reduced environmental tobacco smoke exposure for non-smokers, with the largest benefits for more deprived non-smokers with high levels of exposure. This group were most at risk from the negative externalities of smoking, and the ban was therefore not only an effective but an equitable intervention. Nevertheless, there might still have been a negative equity impact of the ban across the whole population due to an increased gap in exposure between smokers (who are more likely to be deprived) and non-smokers (who are more likely to be affluent). However, our results show that gaps in overall levels of tobacco smoke exposure narrowed between affluent and deprived non-smokers after the ban and bivariate inequality across the whole population did not increase.

For peer review only

ACKNOWLEDGEMENTS

We would like to thank Richard Cookson, Owen O'Donnell, Elena Ratschen, Kamran Siddiqi and Tom Van Ourti for their helpful comments and enlightening debate.

FUNDING

The study was funded by the Wellcome Trust (205427/Z/16/Z). All errors remain our own.

COMPETING INTERESTS

The authors have no competing interests to declare.

AUTHOR CONTRIBUTIONS

Authors contributed equally to the paper. JL extracted the data, performed initial analysis and wrote the first draft. MR developed the methods, performed the final analysis and wrote up the methods, results and appendices. TD wrote the abstract, introduction and discussion. All authors critically edited the manuscript.

DATA AVAILABILITY

Data are from the Health Survey for England, 2007 Wave. This is accessible through the UK Data Service, persistent identifier: 10.5255/UKDA-SN-6112-1. This data is safeguarded, and accessible through an end user license.

For peer review only

REFERENCES

[1] Doll, Richard and Bradford Hill, Austin. "Smoking and carcinoma of the lung". In: *British Medical Journal* 2.4682 (1950), pp. 739–738.

[2] Wald, Nicholas J and Nicolaides-Bouman, Ans. *UK smoking statistics*. Oxford University Press, 1991.

[3] Garfinkel, Lawrence. "Time trends in lung cancer mortality among nonsmokers and a note on passive smoking". In: *JNCI: Journal of the National Cancer Institute* 66.6 (1981), pp. 1061–1066.

[4] Hirayama, Takeshi. "Non-smoking wives of heavy smokers have a higher risk of lung cancer: a study from Japan." In: *BMJ (Clin Res Ed.)* 282.6259 (1981), pp. 183–185.

[5] Trichopoulos, Dimitrios et al. "Lung cancer and passive smoking". In: *International Journal of Cancer* 27.1 (1981), pp. 1–4.

[6] De Waard, F et al. "Urinary cotinine and lung cancer risk in a female cohort". In: *British Journal of Cancer* 72.3 (1995), p. 784.

[7] Jee, Sun Ha, Ohrr, Heechoul, and Kim, Il Soon. "Effects of husbands' smoking on the incidence of lung cancer in Korean women." In: *International Journal of Epidemiology* 28.5 (1999), pp. 824–828.

[8] Nishino, Yoshikazu et al. "Passive smoking at home and cancer risk: a population-based prospective study in Japanese nonsmoking women". In: *Cancer Causes & Control* 12.9 (2001), pp. 797–802.

[9] Fontham, Elizabeth TH et al. "Environmental tobacco smoke and lung cancer in nonsmoking women: a multicenter study". In: *Jama* 271.22 (1994), pp. 1752–1759.

[10] Wang, Longde et al. "Lung cancer and environmental tobacco smoke in a non-industrial area of China". In: *International Journal of Cancer* 88.1 (2000), pp. 139–145.

[11] Hackshaw, Allan K, Law, Malcolm R, and Wald, Nicholas J. "The accumulated evidence on lung cancer and environmental tobacco smoke". In: *BMJ* 315.7114 (1997), pp. 980–988.

For peer review only

[12] Health, US Department of, Services, Human, et al. *The health consequences of involuntary exposure to tobacco smoke: a report of the Surgeon General.* 2006.

[13] Jaakkola, Maritta S and Samet, Jonathan M. "Summary: workshop on health risks attributable to ETS exposure in the workplace." In: *Environmental Health Perspectives* 107.suppl 6 (1999), pp. 823–828.

[14] Jamrozik, Konrad. "Estimate of deaths attributable to passive smoking among UK adults: database analysis". In: *Bmj* 330.7495 (2005), p. 812.

[15] Jha, Prabhat et al. "Social inequalities in male mortality, and in male mortality from smoking: indirect estimation from national death rates in England and Wales, Poland, and North America". In: *The Lancet* 368.9533 (2006), pp. 367–370.

[16] Fowkes, Freya JI et al. "Scottish smoke-free legislation and trends in smoking cessation". In: *Addiction* 103.11 (2008), pp. 1888–1895.

[17] Lee, John, Glantz, Stanton A, and Millett, Christopher. "Effect of smoke-free legislation on adult smoking behaviour in England in the 18 months following implementation". In: *PloS One* 6.6 (2011), e20933.

[18] Jones, Andrew et al. "Do public smoking bans have an impact on active smoking? Evidence from the UK". In: *Health Economics* 24.2 (2015), pp. 175–192.

[19] England, Health Survey for. *Smoking*. Available at http://healthsurvey.hscic.gov.uk/data-visualisation/data-visualisation/explore-the-trends/ smoking.aspx (2019/09/06).

[20] Semple, Sean et al. "UK smoke-free legislation: changes in PM2. 5 concentrations in bars in Scotland, England, and Wales". In: *Annals of occupational hygiene* 54.3 (2010), pp. 272–280.

[21] King, Brian et al. "Socioeconomic variation in the prevalence, introduction, retention, and removal of smoke-free policies among smokers: findings from the International Tobacco Control (ITC) Four Country Survey". In: *International journal of environmental research and public health* 8.2 (2011), pp. 411–434.

[22] Benowitz, Neal L. "Cotinine as a biomarker of environmental tobacco smoke exposure".

For peer review only

In: *Epidemiologic reviews* 18.2 (1996), pp. 188–204.

[23] Jarvis, Martin J. et al. "Assessing smoking status in children, adolescents and adults: cotinine cut-points revisited". In: *Addiction* 103.9 (2008), pp. 1553–1561.

[24] Noble, Michael, Wilkinson, Ms Emma, Barnes, Miss Helen, et al. "The English indices of deprivation 2007". In: (2008).

[25] Rubin, Donald B. "Estimating causal effects of treatments in randomized and nonrandomized studies." In: *Journal of Educational Psychology* 66.5 (1974), p. 688.

[26] Koenker, Roger and Bassett Jr, Gilbert. "Regression quantiles". In: *Econometrica: Journal of the Econometric Society* (1978), pp. 33–50.

[27] Frandsen, Brigham R, Frolich, Markus, and Melly, Blaise. "Quantile treatment effects in the regression discontinuity design". In: *Journal of Econometrics* 168.2 (2012), pp. 382–395.

[28] Firpo, Sergio and Pinto, Cristine. "Identification and estimation of distributional impacts of interventions using changes in inequality measures". In: *Journal of Applied Econometrics* 31.3 (2016), pp. 457–486.

[29] Kakwani, Nanak C. "Measurement of tax progressivity: an international comparison". In: *The Economic Journal* 87.345 (1977), pp. 71–80.

[30] Heckley, Gawain, Gerdtham, Ulf-G, and Kjellsson, Gustav. "A general method for decomposing the causes of socioeconomic inequality in health". In: *Journal of health economics* 48 (2016), pp. 89–106.

[31] Layte, Richard and Whelan, Christopher T. "Explaining social class inequalities in smoking: the role of education, self-efficacy, and deprivation". In: *European Sociological Review* 25.4 (2008), pp. 399–410.

[32] Borland, Ron et al. "Determinants and consequences of smoke-free homes: findings from the International Tobacco Control (ITC) Four Country Survey". In: *Tobacco control* 15.suppl 3 (2006), pp. iii42–iii50.

For peer review only

[33] Brown, Tamara, Platt, Stephen, and Amos, Amanda. "Equity impact of population level interventions and policies to reduce smoking in adults: a systematic review". In: *Drug and alcohol dependence* 138 (2014), pp. 7–16.

[34] Sims, Michelle et al. "Did smokefree legislation in England reduce exposure to secondhand smoke among nonsmoking adults? Cotinine analysis from the Health Survey for England". In: *Environmental health perspectives* 120.3 (2011), pp. 425–430.

[35] Robson, Matthew, Doran, Tim, and Cookson, Richard. "Estimating and Decomposing Conditional Average Treatment Effects: The Smoking Ban in England". In: *HEDG Working Papers* 19.20 (2019).

[36] Adda, Jerome and Cornaglia, Francesca. "The effect of bans and taxes on passive smoking". In: *American Economic Journal: Applied Economics* 2.1 (2010), pp. 1–32.

For peer review only

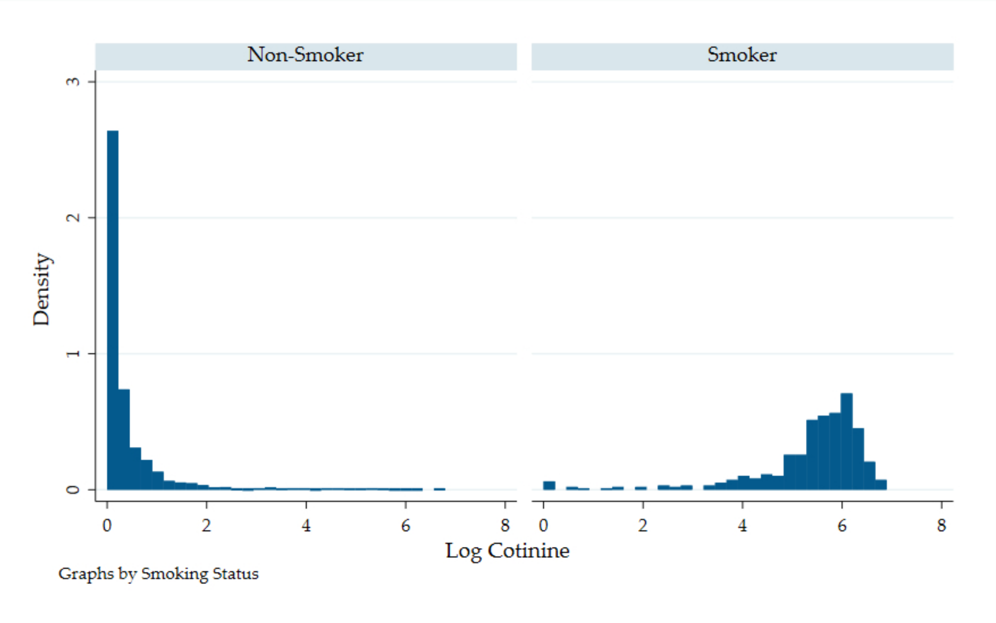

Figure 1: Distribution of Log Cotinine: Non-Smokers and Smokers

310x194mm (72 x 72 DPI)

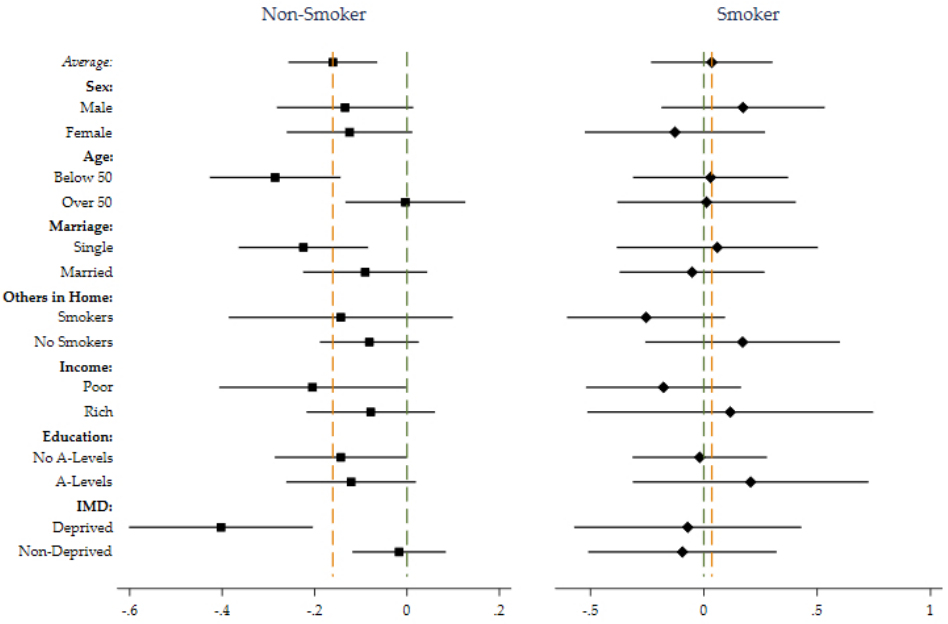

Figure 2: Forest Plot of CATEs of Log Cotinine: Non-Smokers and Smokers

267x194mm (72 x 72 DPI)

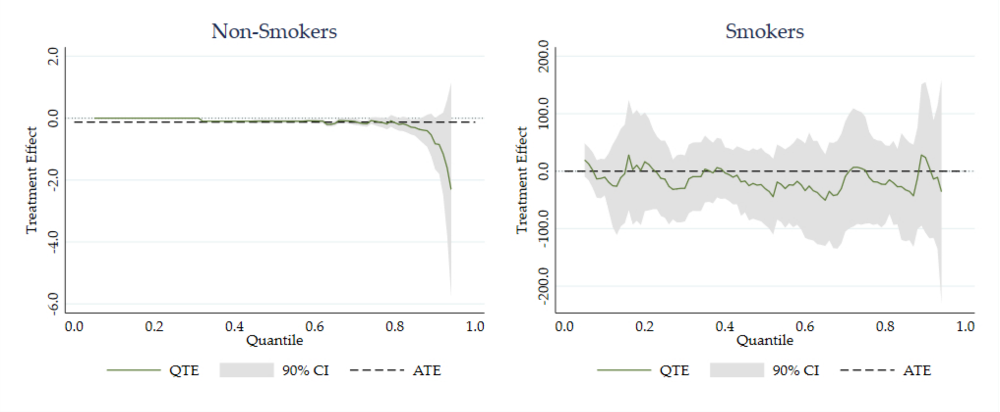

Figure 3: Quantile Treatment Effects: Log Cotinine

358x149mm (72 x 72 DPI)

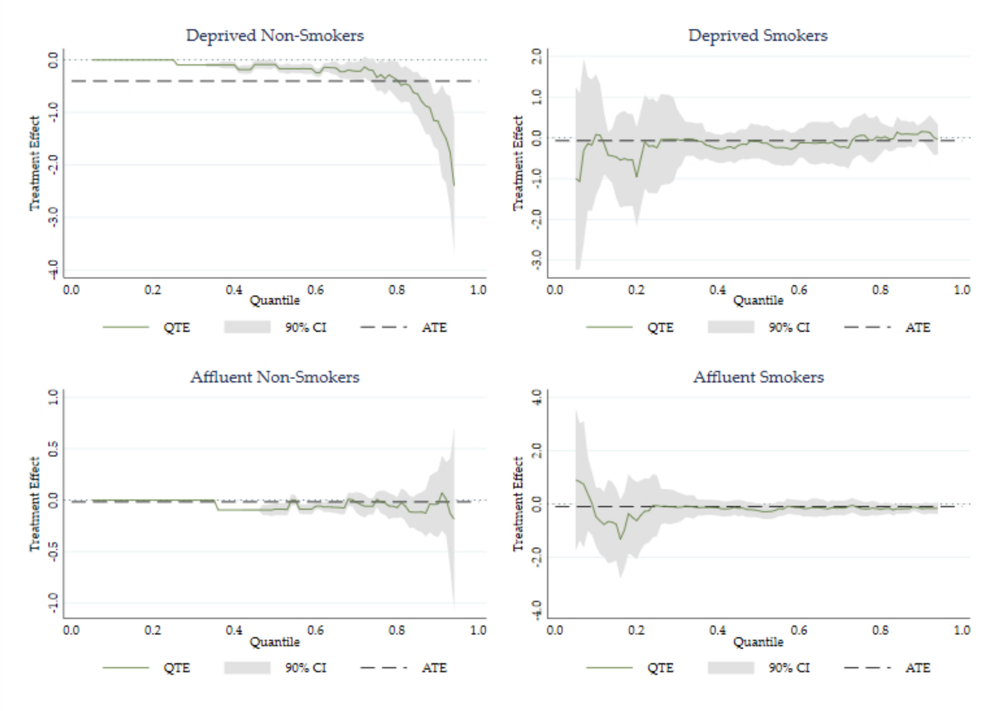

Figure 4: Conditional Quantile Treatment Effects: Log Cotinine

271x194mm (72 x 72 DPI)

A Appendix

A.1 Sensitivity: Cotinine

Throughout the paper we use log(cotinine +1) as our outcome variable. We use this transformed variable due to the highly right skewed nature of cotinine and the potential impact of extreme values on results. Here we present the main results using cotinine, rather than log cotinine. Throughout results are robust to the choice of variable, but standard errors tend to increase for cotinine.

As in the main analysis, for the average treatment effect there are large and significant reductions for non-smokers, and no significant reductions for smokers.

Table 2: Cotinine: Regression Discontinuity

	(1)<br>**Non-Smokers**<br>Coef./S.E.	(2)<br>**Smokers**<br>Coef./S.E.
Treatment	-4.9852**	-12.1932
	(2.5121)	(32.1071)
Time	0.0016	-0.0230
	(0.0293)	(0.2154)
Treatment X Time	0.0501	-0.0729
	(0.0335)	(0.3449)
**Constant**	5.3445**	301.0218***
	(2.4040)	(18.6132)
N	2916	759
R-squared	0.0026	0.0030

* $p < 0.10$, ** $p < 0.05$, *** $p < 0.01$

For conditional average treatment effects, we observe no significant effects for smokers conditional on any variable. For non-smokers, we observe both significant effects and differences between subgroups. The difference between age groups here disappears and standard errors do increase in comparison to the main results. When we estimate these effects using negative binomial regressions, however, these standard errors reduce.

For peer review only

Figure 5: Forest Plot of CATEs of Cotinine: Non-Smokers and Smokers

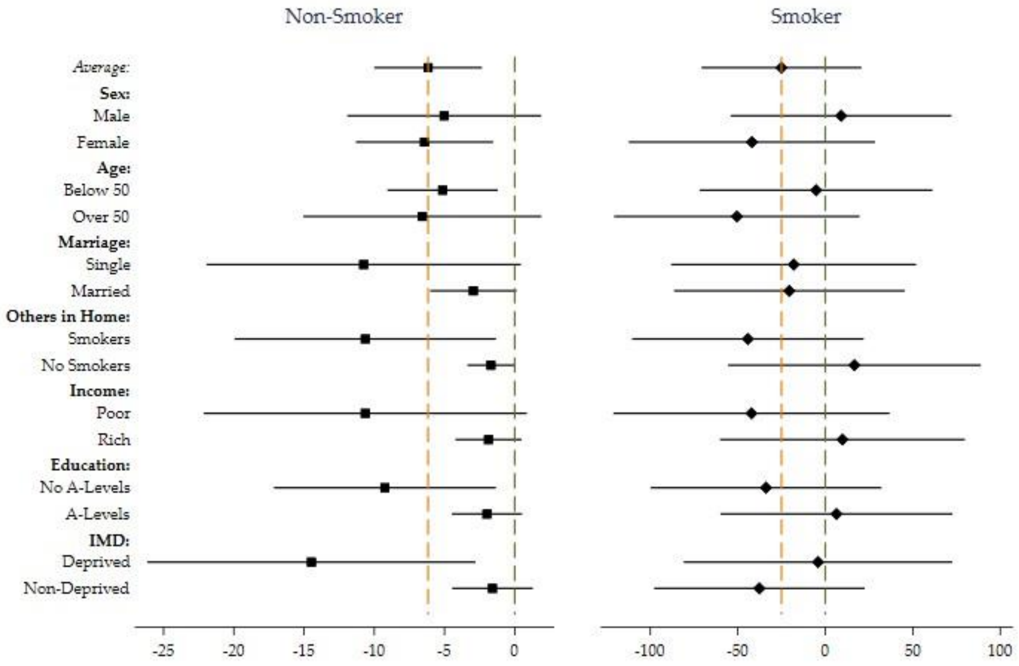

Quantile treatment effects are similar in significance and direction to the main results. There are significant, and increasing, treatment effects for higher quantiles for non-smokers and no significant effects for smokers, at any quantile.

Figure 6: Quantile Treatment Effects: Cotinine

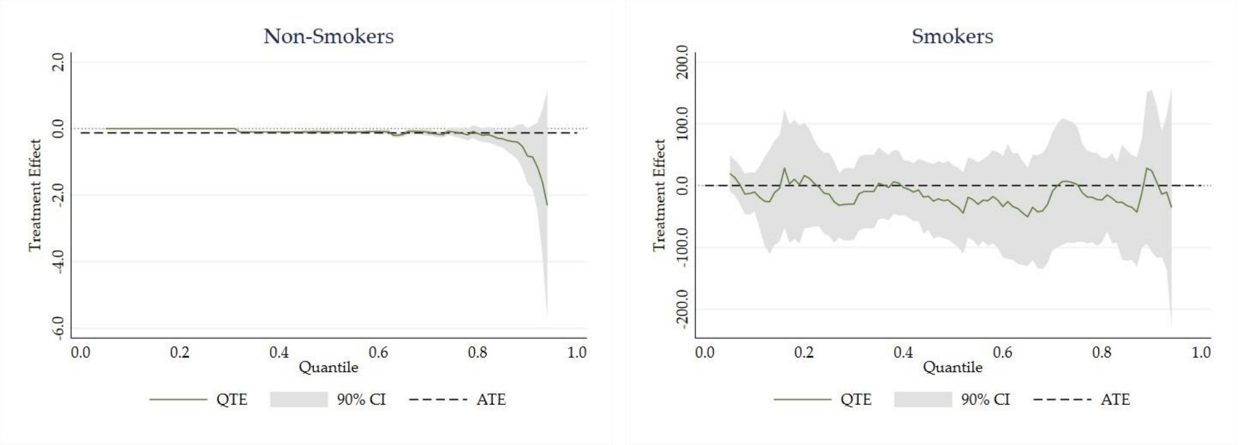

For peer review only - http://bmjopen.bmj.com/site/about/guidelines.xhtml

Inequality treatment effects are very similar to the main results. The concentration of cotinine amongst deprived non-smokers was significantly reduced, whilst no significant effects were observed at the sample level or for smokers.

Table 3: Cotinine: Concentration Index

	(1)	(2)	(3)
	**Sample**	**Non-Smokers**	**Smokers**
	Coef./S.E.	Coef./S.E.	Coef./S.E.
Treatment	-0.0209	0.5571***	-0.0728
	(0.1031)	(0.1647)	(0.0648)
Time	0.0011	-0.0062**	0.0006
	(0.0008)	(0.0024)	(0.0004)
Treatment X Time	-0.0018	0.0045	-0.0007
	(0.0012)	(0.0031)	(0.0007)
**Constant**	-0.1335**	-0.5714***	0.0356
	(0.0609)	(0.1835)	(0.0379)
N	3675	2916	759
R-squared	0.0010	0.0026	0.0060

* $p < 0.10$, ** $p < 0.05$, *** $p < 0.01$

For peer review only

A.2 Covariate Balance and Weights

A.2.1 Covariate Balance

Central to the estimation of treatment effects is the comparability of characteristics of the control and treated groups. If there are imbalances in covariates the estimated effects may be biased. To test if there are significant differences in covariates, $x_j, \ \forall j \in J$, the covariate of interest can be regressed against the treatment dummy, *D*:

$$\boldsymbol{x_j} = \beta_0 + \boldsymbol{D}\beta_1 + \boldsymbol{u} \tag{8}$$

Here β_1 identifies the extent and significance of covariate imbalance between control and treated groups. In regression discontinuity designs, however, the crucial assumption is that there are no significant discontinuities in covariates at the *threshold*. Imbalances between control and treated groups *on average* are not important if this assumption holds. In a simple linear framework, this assumption can be tested by regressing the covariates against the treatment dummy, *D*, (centred) forcing variable, *Z* and their interaction:

$$\boldsymbol{x}_j = \gamma_0 + \boldsymbol{D}\gamma_1 + \boldsymbol{Z}\gamma_2 + (\boldsymbol{D}_0 \times \boldsymbol{Z})\gamma_3 + \boldsymbol{u}, \qquad for\ |\boldsymbol{Z}| \leq h \tag{9}$$

If γ_1 is significant it shows there is a discontinuity in $\boldsymbol{x}_j$ at the threshold. This imbalance at the threshold could bias the estimation of the treatment effect.

A.2.2 Inverse Probability of Treatment Weights

To mitigate any potential biases that these imbalances could cause, doubly-robust methods which use propensity weights alongside the regression discontinuity design, can be used. We estimate the inverse probability of treatment to use as weights. This balances covariates across treatment groups, giving lower weight to those characteristics which are predictors of treatment (i.e. via selection into treatment).

For peer review only - http://bmjopen.bmj.com/site/about/guidelines.xhtml

To estimate propensity weights the following regression is run, as a logit:

$$\boldsymbol{D} = \delta_0 + \sum_{j}^{J} \left(\boldsymbol{X_j} \delta_{1j} + \left(\boldsymbol{X_j'} \times |\boldsymbol{Z}| \right) \delta_{2j} \right) + \boldsymbol{u} \qquad for\ |\boldsymbol{Z}| \leq h \tag{10}$$

This formulation includes both covariates $\boldsymbol{X_j}$ and the interaction between those covariates and the absolute value of the forcing variable **Z**. This particular formulation is used to ensure balance not just in expectation (as in Equation (8)) but also at the discontinuity. By using the absolute value of **Z** we provide this flexibility, whilst ensuring the overlap assumption is not violated. These weights are estimated separately for each subgroup.

Using this we predict the probability of being treated, given the observable characteristics $Pr(D = 1|X, Z)$. The inverse probability of treatment weights are then calculated as:

$$w_i^{IPT} = \frac{D}{\Pr\left(D = 1\ |X, Z\right)} + \frac{1 - D}{1 - \Pr\left(D = 1\ |X, Z\right)} \tag{11}$$

Table 4 shows the consequence of using these weights on covariate balance. Each coefficient (and standard error) are results from separate regressions, with the outcome variable x_j in the first column. Average differences are the β_1 coefficients of Eq.(8), with differences at the discontinuity being the γ_1 coefficients of Eq.(9).

In the unweighted sample we see that the treated group, on average, live in more rural and less deprived areas. At the discontinuity, respondents have significantly fewer children, are more likely to be retired and live in rural areas. These differences could potentially cause bias in the estimates of the treatment effect and need to be controlled for. When the weights are applied, however, we observe no significant differences either on average or at the discontinuity; removing the selection bias (on observables) which could have biased estimates.

For peer review only - http://bmjopen.bmj.com/site/about/guidelines.xhtml

Table 4: Balance

	Unweighted		Weighted	
	(1)	(2)	(3)	(4)
	**Average**	**Discontinuity**	**Average**	**Discontinuity**
	Coef./Std. err.	Coef./Std. err.	Coef./Std. err.	Coef./Std. err.
Age	0.6179	3.5169***	-0.0543	-0.2464
	(0.5288)	(1.0511)	(0.6107)	(1.1586)
Sex (1 = female)	0.0025	-0.0051	0.0005	0.0019
	(0.0149)	(0.0297)	(0.0175)	(0.0337)
Married Couple	-0.0155	-0.0023	-0.0004	0.0026
	(0.0148)	(0.0296)	(0.0173)	(0.0331)
Number of Children	-0.0248	-0.2123***	-0.0007	0.0145
	(0.0239)	(0.0445)	(0.0298)	(0.0592)
Retired	0.0153	0.0829***	-0.0016	-0.0038
	(0.0132)	(0.0266)	(0.0149)	(0.0283)
Ethnicity (1 = white)	0.0170**	0.0089	-0.0023	-0.0056
	(0.0085)	(0.0166)	(0.0106)	(0.0207)
Urban	-0.0088	-0.1027***	0.0034	0.0069
	(0.0126)	(0.0249)	(0.0141)	(0.0254)
IMD Quintile (1 = deprived)	0.0880**	0.1039	-0.0013	-0.0064
	(0.0418)	(0.0838)	(0.0496)	(0.0960)
Occupation: Manager	0.0065	0.0202	-0.0017	-0.0085
	(0.0142)	(0.0284)	(0.0165)	(0.0316)
Education: A-Level	0.0183	-0.0047	-0.0023	-0.0040
	(0.0149)	(0.0297)	(0.0175)	(0.0336)
Home Owner	0.0419***	-0.0038	0.0024	0.0043
	(0.0130)	(0.0259)	(0.0152)	(0.0289)

* $p < 0.10$, ** $p < 0.05$, *** $p < 0.01$

A.2.3 Triangular Kernels

We run each regression discontinuity design as a locally-weighed regression, using triangular kernels to give higher weights to observations closer to the threshold. These weights are calculated as:

$$w_i^{TRI} = h - |Z_i|$$

The highest weights are at the threshold ($Z = 0$) and decrease linearly as the bound of the bandwidth, h is reached. These are multiplied by the inverse probability of treatment weights to provide the final weights ($w_i = w_i^{IPT} . w_i^{TRI}$) for the weighted regressions run:

$$\sqrt{w_i}\, \boldsymbol{y} = \sqrt{w_i}\,(\beta_0 + \boldsymbol{D}\beta_1 + \boldsymbol{Z}\beta_2 + (\boldsymbol{D} \times \boldsymbol{Z})\beta_3 + \boldsymbol{u}) \qquad for\ |\boldsymbol{Z}| \leq h \qquad (12)$$

For peer review only - http://bmjopen.bmj.com/site/about/guidelines.xhtml

A.3 Baseline Characteristics

Table 5 summarises the baseline characteristics of the control group, by cotinine quintile. Smoking variables are strongly associated with cotinine, with higher numbers of smokers, higher intensity of smoking and higher exposure to smoke at higher cotinine quintiles. Those with lower levels of cotinine are older, more likely to be female, married and retired, to live in rural areas and have more children. Higher socioeconomic status individuals have lower levels of cotinine, for a range of variables: IMD, income, occupation, education and home ownership. Additionally, those with higher cotinine levels have lower self-assessed health, more limiting longstanding illnesses and they drink more heavily.

For peer review only

Table 5: Descriptive Statistics by Cotinine Quintile

	**Cotinine Quintile**					**Summary**
	**Q1**	**Q2**	**Q3**	**Q4**	**Q5**	**Mean**
	Mean	Mean	Mean	Mean	Mean	Mean
**Cotinine Levels**						
Cotinine	0.00	0.14	0.38	7.61	338.18	69.13
Log Cotinine	0.00	0.13	0.32	1.37	5.68	1.47
**Smoking**						
Smoker	0.01	0.00	0.00	0.12	0.93	0.21
Packs Smoked Per Day	0.00	0.00	0.00	0.14	2.40	0.51
Exposed to Smoke	0.35	0.33	0.44	0.60	0.72	0.48
Hours Exposed to Smoke	1.22	1.18	2.95	7.65	12.37	4.89
Ever Smoked	0.45	0.49	0.54	0.61	0.99	0.61
Smoke in Pubs	0.00	0.00	0.00	0.06	0.28	0.07
Exposed in Pubs	0.16	0.16	0.25	0.38	0.38	0.26
**Demographics**						
Age	52.18	52.35	50.11	48.23	44.80	49.70
Sex (1 = female)	0.54	0.56	0.54	0.52	0.50	0.53
Married Couple	0.67	0.65	0.65	0.54	0.42	0.59
Number of Children	0.46	0.37	0.47	0.40	0.54	0.44
Retired	0.29	0.31	0.28	0.25	0.11	0.25
Ethnicity (1 = white)	0.90	0.91	0.89	0.90	0.92	0.91
Urban	0.76	0.72	0.71	0.80	0.84	0.77
**Socioeconomic Status**						
IMD Quintile (1 = deprived)	3.47	3.44	3.25	2.94	2.62	3.16
Log Equivalised Income	10.14	10.19	10.17	10.01	9.79	10.06
Occupation: Manager	0.42	0.41	0.38	0.31	0.22	0.35
Education: A-Level	0.50	0.49	0.49	0.44	0.33	0.45
Home Owner	0.84	0.78	0.78	0.73	0.54	0.74
**Health Variables**						
Self Assessed Health (5 = v. good)	4.09	4.18	4.08	3.89	3.68	3.99
Limiting Longstanding Illness	0.27	0.23	0.25	0.26	0.33	0.27
Alcohol Units: Heaviest Day	5.34	5.42	6.88	7.81	9.49	6.89

For peer review only - http://bmjopen.bmj.com/site/about/guidelines.xhtml

A.4 Average Treatment Effects

Table 6 shows the results from the regression discontinuity design. The average treatment effect of the ban on non-smokers is large and significant, at -0.128, whilst there is no significant effect for smokers. The constant shows the expected level of log cotinine exposure at the time of the ban, for the control group: 0.399 for non-smokers and 5.343 for smokers.

Table 6: Log Cotinine: Regression Discontinuity

	(1) Non-Smokers Coef./S.E.	(2) Smokers Coef./S.E.
Treatment	-0.1279**	0.0385
	(0.0632)	(0.1662)
Time	-0.0004	-0.0001
	(0.0005)	(0.0012)
Treatment X Time	0.0010	-0.0006
	(0.0008)	(0.0018)
**Constant**	0.3987***	5.3434***
	(0.0395)	(0.1089)
N	2952	766
R-Squared	0.0062	0.005

* $p < 0.10$, ** $p < 0.05$, *** $p < 0.01$

Figure 7 shows the regression discontinuity plot for smokers and non-smokers. This shows the (weighted) mean log cotinine (averaged across two days) plotted against the number of days from the ban. The line plot shows the predicted point estimates from the regression of Table 6, whilst the area plot shows the 90% confidence intervals. We observe a discontinuity at the time of the ban for non-smokers, but not smokers. These are the average treatment effects observed in Table 6.

This figure is indicative of the quantile treatment effects we observe in Figure 3: many observations at the lower values of log cotinine move the small distance towards zero, whilst those higher values of log cotinine become much less frequent following the ban.

For peer review only - http://bmjopen.bmj.com/site/about/guidelines.xhtml

Figure 7: Regression Discontinuity Plot: Non-Smokers and Smokers

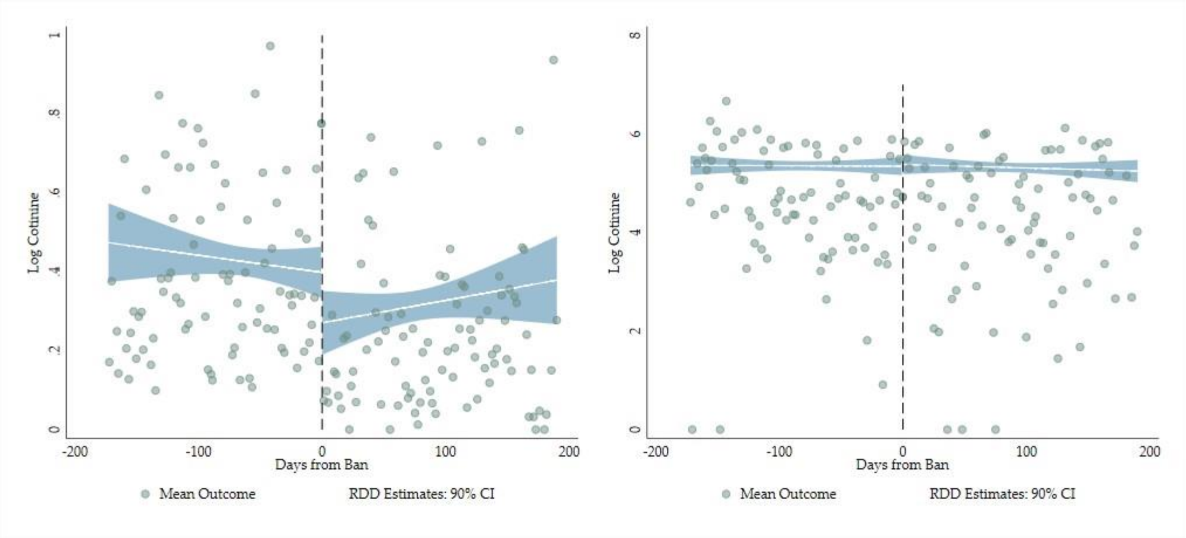

For peer review only

A.5 Sensitivity Analysis

Figure 8 shows a forest plot of sensitivity analysis for the estimated average treatment effects. The baseline point estimate of Table 3 is shown at the top, with 90% confidence intervals, for non-smokers and smokers. The estimates below show how sensitive these results are to changes in the functional form of the regression discontinuity, the size of the bandwidth, the weights used and to the addition of controls.

Figure 8: Sensitivity Forest Plot: Log Cotinine, Non-Smokers and Smokers

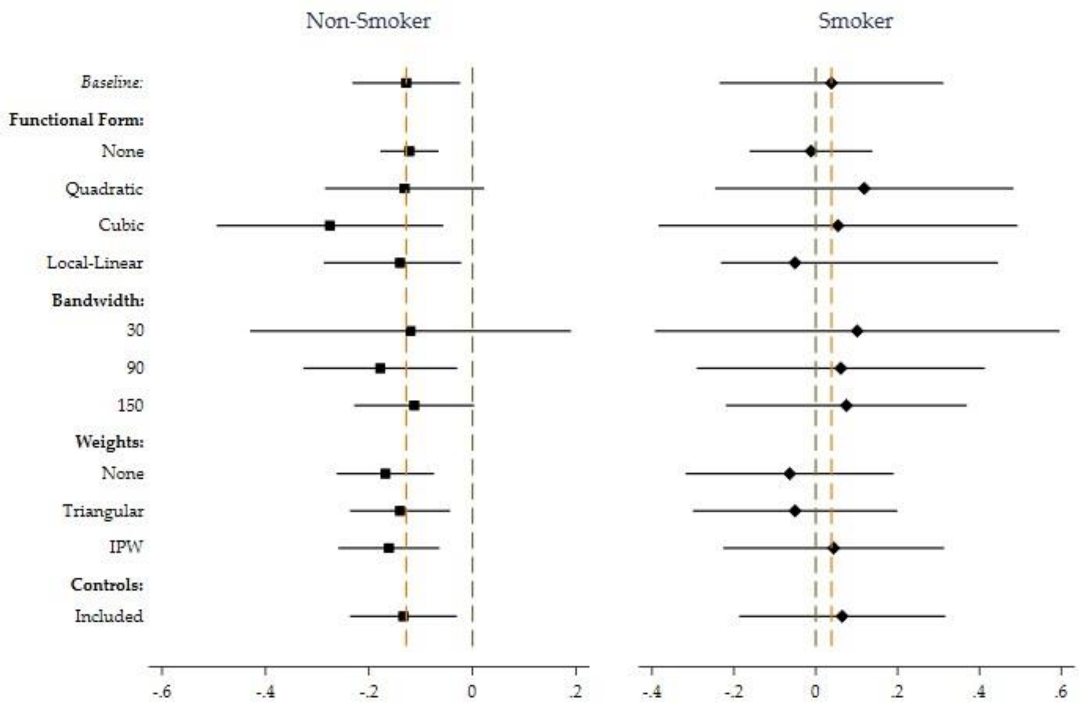

Results show that the analysis appears robust to each of these sensitivity checks. For non-smokers, the effects are never significantly different from the baseline results (dotted orange line) and (with exception to the use of a small bandwidth and quadratic functional form) the estimated effects are always significant, at the 90% level. For smokers, the effects are never significantly different from the baseline effect nor are they significantly different from zero, and indeed appear to hover around a zero effect.

For peer review only - http://bmjopen.bmj.com/site/about/guidelines.xhtml

A.6 Predicted Conditional Potential Outcomes

Figure 9 plots the predicted potential outcomes conditional on observable characteristics. This shows the expected levels of log cotinine at the threshold, for control (red) and treated (blue) groups. These results should be used to supplement the estimated treatment effects in Figure 2; the treatment effects are the differences between these potential outcomes.

Figure 9: Forest Plot of Potential Outcomes of Log Cotinine: Non-Smokers and Smokers

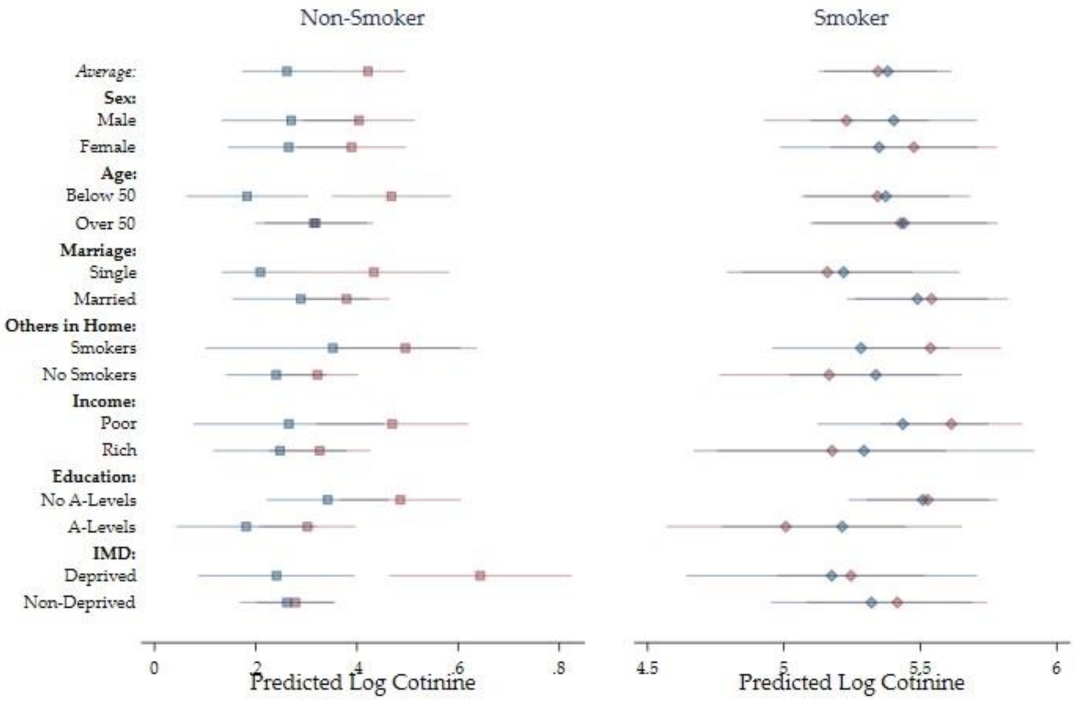

Non-smokers are found to have both significant differences between control and treated potential outcomes (i.e. significant treatment effects) and are found to have differences in exposure levels between subgroups. After the ban the differences between those subgroups that are different before, is much reduced. For smokers, there are no significant differences between potential outcomes; neither between control and treated, nor between subgroups.

For peer review only - http://bmjopen.bmj.com/site/about/guidelines.xhtml

BMJ Open

Estimating the Equity Impacts of the Smoking Ban in England on Cotinine Levels: A Regression Discontinuity Design

Journal:	*BMJ Open*
Manuscript ID	bmjopen-2021-049547.R1
Article Type:	Original research
Date Submitted by the Author:	21-Jul-2021
Complete List of Authors:	Robson, Matthew; University of York Department of Health Sciences, Department of Health Sciences<br>Lord, Joseph; University of York Centre for Health Economics, Centre for Health Economics<br>Doran, Tim; University of York Department of Health Sciences, Department of Health Sciences
**Primary Subject Heading**:	Research methods
Secondary Subject Heading:	Health policy, Public health, Health economics, Smoking and tobacco
Keywords:	HEALTH ECONOMICS, PUBLIC HEALTH, STATISTICS & RESEARCH METHODS

SCHOLARONE™
Manuscripts

I, the Submitting Author has the right to grant and does grant on behalf of all authors of the Work (as defined in the below author licence), an exclusive licence and/or a non-exclusive licence for contributions from authors who are: i) UK Crown employees; ii) where BMJ has agreed a CC-BY licence shall apply, and/or iii) in accordance with the terms applicable for US Federal Government officers or employees acting as part of their official duties; on a worldwide, perpetual, irrevocable, royalty-free basis to BMJ Publishing Group Ltd ("BMJ") its licensees and where the relevant Journal is co-owned by BMJ to the co-owners of the Journal, to publish the Work in this journal and any other BMJ products and to exploit all rights, as set out in our licence.

The Submitting Author accepts and understands that any supply made under these terms is made by BMJ to the Submitting Author unless you are acting as an employee on behalf of your employer or a postgraduate student of an affiliated institution which is paying any applicable article publishing charge ("APC") for Open Access articles. Where the Submitting Author wishes to make the Work available on an Open Access basis (and intends to pay the relevant APC), the terms of reuse of such Open Access shall be governed by a Creative Commons licence – details of these licences and which Creative Commons licence will apply to this Work are set out in our licence referred to above.

Other than as permitted in any relevant BMJ Author's Self Archiving Policies, I confirm this Work has not been accepted for publication elsewhere, is not being considered for publication elsewhere and does not duplicate material already published. I confirm all authors consent to publication of this Work and authorise the granting of this licence.

For peer review only

Estimating the Equity Impacts of the Smoking Ban in England on Cotinine Levels: A Regression Discontinuity Design

Matthew Robson[1], Joseph Lord[2] & Tim Doran[3]

July 13th, 2021

For peer review only

Abstract

Objective. To estimate the equity impacts of the 2007 smoking ban in England, for both smokers and non-smokers.

Design. Doubly-robust regression discontinuity analysis of salivary cotinine levels. Conditional average treatment effects were used to estimate differential impacts of the ban by socioeconomic deprivation (based on the Index of Multiple Deprivation). Distributional impacts were further assessed using conditional quantile treatment effects and inequality treatment effects.

Setting. In 2007, England introduced a ban on smoking in public places. This had little impact on tobacco consumption by smokers, but was associated with decreases in environmental tobacco smoke exposure for non-smokers. However, the impact of the ban on socioeconomic inequalities in exposure are unclear.

Participants. 766 smokers and 2,952 non-smokers responding to the Health Survey for England in 2007.

Outcome Measure. Levels of salivary cotinine.

Results. Before the ban, socioeconomic deprivation was associated with higher cotinine levels for non-smokers, but not for smokers. The ban caused a significant reduction in average cotinine levels for non-smokers (p=0.043), but had no effect for smokers (p=0.817). Reductions for non-smokers were greater for more deprived groups with higher levels of exposure, and there was a significant reduction in socioeconomic-related inequality in cotinine. Across the whole population (both smokers and non-smokers), there was no significant increase in the concentration of cotinine levels amongst the socioeconomically deprived.

Conclusion. The 2007 ban on smoking in public places had little impact on smokers, but was, as intended, associated with reductions in both i) average levels of environmental

[1] Department of Health Sciences, University of York, York, United Kingdom. Email: matthew.robson@york.ac.uk.
[2] Centre for Health Economics, University of York, York, United Kingdom. Email: josephlord534@gmail.com.
[3] Corresponding author: Tim Doran, Address: Department of Health Sciences, University of York, Heslington, York, United Kingdom, YO10 5DD. Email: tim.doran@york.ac.uk. Website: https://equipol.org. Phone: 01904 321524.

tobacco smoke exposure, and ii) deprivation-related inequality in exposure among non-smokers.

Keywords: Health Economics, Public Health, Statistics and Research Methods.

Wordcount: 3044

Strengths and limitations of this study

- We used a doubly-robust regression discontinuity design to derive causal estimates of the equity impacts of the smoking ban for both smokers and non-smokers.
- Data were derived from a rich, individual-level dataset with an objective measure of tobacco smoke exposure to avoid social desirability bias in reported variables.
- Causal effects of the ban were estimated across the entire distribution of the outcome, identifying conditional distributional effects and the impact on socioeconomic-related inequality.
- We focused only upon the short-term effects of the ban and rely on an area-based measure of socioeconomic status.

For peer review only

INTRODUCTION

Successive UK governments have pursued a range of public health policies to reduce population harms from tobacco smoke exposure since links to lung cancer and other common fatal conditions were confirmed in the 1950s[1]. With public education, increased taxation and restrictions on sales and advertising, smoking prevalence fell from 70% in the 1950s to under 25% percent by the turn of the century[2]. More coercive policies began to be considered in the 1990s as studies emerged identifying harmful impacts on non-smokers exposed to environmental smoke[3-12], including exposure at home[7,8] and in the workplace[13]. Estimates of the population impact of environmental exposure suggest that over 10 percent of smoking-related deaths may occur in non-smokers[14], and the risks to smokers themselves are further increased by environmental smoke. The impacts of smoking are also unevenly distributed; whilst rates of smoking have fallen in all socioeconomic groups since the 1950s, they fell fastest in the most affluent groups. At the end of the century, over half of the difference in mortality between men in high (professional, managerial and technical) and low (unskilled manual) socioeconomic groups was attributable to tobacco exposure[15].

The growing weight of evidence on environmental tobacco smoke eventually led to the implementation of national bans in Scotland (March 2006), Wales and Northern Ireland (April 2007), and England (July 2007). For England and Wales, the Health Act 2006 effectively prohibited tobacco smoking in enclosed public places, including workplaces, public transport and private clubs. The ban was found to have little impact on tobacco consumption in the short run [16-20], but smokers were found to smoke substantially less in enclosed public places, including at work and in pubs[17].

Evidence on the equity impacts of the smoking ban, particularly for non-smokers, is less clear. In England, smoking prevalence continued to fall at similar rates across different socioeconomic status (SES) groups after the ban, preserving the gaps between them; by 2015 smoking prevalence remained above 25% in the most deprived fourth and fifth of areas, compared with 10% in the least deprived fifth[19]. In terms of second-hand exposure, Semple et al. noted that pubs in more deprived areas, with higher baseline particulate (PM2.5) concentrations, tended to have greater reductions after the ban[21]. In contrast, King et al. found that higher SES smokers were more likely than low status smokers to prohibit smoking in their own home following the ban[22].

For peer review only

Obtaining more direct evidence on the equity impacts of smoking bans on non-smokers is subject to two key limitations: a reliance on before-and-after study designs measuring association, rather than strong econometric designs identifying causal effects; and a focus on average effects for the whole population, rather than distributional effects relating to baseline exposure to tobacco smoke and socioeconomic circumstances. The latter limitation is particularly important, as national policies of this kind are frequently less effective for less affluent socioeconomic groups with higher baseline levels of exposure, and this has the unintended consequence of widening existing health inequalities. In this paper, we assess the impact of the national smoking ban on cotinine levels, a biomarker of tobacco smoke exposure, for both smokers and non-smokers. We use approaches that assess the full range of effects in relation to socioeconomic characteristics across the entire distribution of exposure levels, to more accurately determine the equity impacts of the smoking ban.

METHODS

Intervention

The national smoking ban in England was implemented on 1st July 2007. Smoking in enclosed public places became an offence, subject to fines of up to £200 for individuals and £2,500 for businesses. Exemptions were included, at that time, for psychiatric units, nursing homes, prisons and other specified public places.

Data

Data were derived from the Health Survey for England (HSE), an annual cross-sectional survey which records health and lifestyle changes over time in England at the individual and household level. The dataset includes variables covering tobacco smoking and exposure, as well as a range of individual characteristics recorded by an interview and, if consent is given, a follow-up nurse's visit (on average 18.4 days later). Our main analysis uses data from the 2007 wave of the survey, from January 2007 to January 2008, covering the period of the introduction of the public smoking ban in England.

For peer review only

Patient and Public Involvement

As we conduct a secondary analysis of survey data, there was no direct patient and public involvement.

Tobacco Exposure

The primary outcome is exposure to tobacco smoke, estimated by salivary cotinine concentration. Cotinine is a metabolite of nicotine which diffuses from the bloodstream into saliva, making salivary cotinine concentration a convenient and reliable biomarker of regular tobacco exposure[23]. Salivary cotinine concentrations above 12ng/ml indicate smoking status with high sensitivity and specificity[24] and scale with the extent of tobacco use. Lower concentrations are also detectable, so cotinine is useful for monitoring changes in both tobacco use by smokers and exposure to second-hand smoke in non-smokers. Distributions of salivary cotinine levels tend to be heavily skewed; for non-smokers, levels are often undetectable whereas for smokers a minority of concentrations are extremely high, and these can have a disproportionate impact on results. We therefore used log(cotinine+1) as our primary outcome, but also report results using untransformed values in Appendix A.1. The subset of respondents to the HSE receiving a nurse visit are asked to provide a saliva sample, which is then analysed for cotinine concentration. For 2007, 4,058 valid cotinine measurements were available.[1] Current smoking status (smoker/non-smoker) was based on self-report.[2]

Socioeconomic deprivation

Our primary measure of socioeconomic deprivation was the 2007 Index of Multiple Deprivation (IMD) quintile[25]. This is an area-based measure linked to postcode, for which we use the quintiles or group into "deprived" (the lowest two quintiles) and "non-deprived" (highest three quintiles). IMD was selected over alternative SES variables, such as household income and occupation, as it had fewer

---

[1] In our main analysis 3,718 responses were used. Of these, 309 were excluded due to the bandwidth of the regression discontinuity design and a further 31 were excluded because observations for control variables were missing.

[2] In the pre-intervention sample, 95.7% of self-reported smokers and 4.3% of non-smokers had cotinine concentrations >12ng/ml. Reporting error on smoking status, therefore, appears low.

For peer review only

missing data, demonstrated the largest differential conditional effect, and was more stable during the period between the main survey and nurse follow-up.

Analysis

Regression Discontinuity Design

We used a sharp regression discontinuity (RD) design to estimate the causal effects of the intervention. The design uses 1st July 2007, the date of implementation of the national smoking ban, as a threshold dividing the sample into 'control' (interviewed before the ban) and 'treated' (interviewed after the ban): with a bandwidth of +/- 190 days. Doubly-robust methods were used, with inverse probability of treatment weights in locally-weighted regressions with triangular kernels, to control for potential covariate imbalances at the time of the ban. More details are provided in Appendix A.2.

The model takes the general form:

$$\boldsymbol{y} = \beta_0 + \boldsymbol{D}\beta_1 + \boldsymbol{Z}\beta_2 + (\boldsymbol{D} \times \boldsymbol{Z})\beta_3 + \boldsymbol{u} \quad for\ |\boldsymbol{Z}| \leq h \#(1)$$

Where **y** is the outcome variable (log cotinine), **D** is the treatment dummy and **Z** is the forcing variable, time from ban, which takes the value zero on the day of the policy change, is positive if the observation is after the ban, and negative before. **u** is the error term, which is assumed to be independent and identically distributed with an expected value of zero. For the coefficients, β_0 represents the average outcome for the control group at the point of discontinuity where $Z \rightarrow 0$ (date of the ban), β_1 is the treatment effect at the discontinuity, β_2 is the time trend before the ban, and β_3 indicates the change in the trend after the ban.

Treatment Effects

We used the RD approach to estimate the potential outcomes individuals would have had with and without treatment at the threshold[26]. The overall impact of the smoking ban on tobacco exposure was measured as an average treatment effect:

For peer review only

$$ATE = \beta_1 = E[\boldsymbol{Y}(1) - \boldsymbol{Y}(0) \mid \boldsymbol{Z} = 0] \#(2)$$

Where Y(0) is the *potential* outcome an individual would have had without treatment and Y(1) is the *potential* outcome with treatment. We then evaluated the distributional impacts of the smoking ban using a series of approaches: i) Conditional Average Treatment Effects ii) Quantile Treatment Effects; iii) Conditional Quantile Treatment Effects; and iv) Inequality Treatment Effects.

Conditional Average Treatment Effects (CATEs) estimate the average effect of the treatment (the smoking ban) on y (log cotinine) conditional on x (e.g. whether the effect of the smoking ban varied with socioeconomic deprivation):

$$CATE(\tilde{\boldsymbol{x}}) = E[\boldsymbol{Y}(1) - \boldsymbol{Y}(0) \mid \tilde{\boldsymbol{X}} = \tilde{\boldsymbol{x}}, \boldsymbol{Z} = 0] \#(3)$$

Quantile Treatment Effects estimate the effect of the treatment on *y* at different quantiles (τ) of *y*:

$$QTE(\tau) = Q^{\tau}_{Y(1)|\boldsymbol{Z}=0} - Q^{\tau}_{Y(0)|\boldsymbol{Z}=0} \#(4)$$

Where $Q^{\tau}_{Y(.)|\boldsymbol{Z}=0}$ is the quantile function which returns the cotinine level Y(.) at the threshold for each quantile, $\tau \in (0,1)$ (conditional rank of the outcome). The differences between these quantile functions gives the treatment effect at each quantile. These effects are estimated directly, using quantile regressions[27] within the RD framework[28]. This allows us to determine how the ban changed the distribution of tobacco exposure, revealing whether levels fell more at the top or bottom of the distribution.

Conditional Quantile Treatment Effects (CQTEs) combine QTEs and CATEs, estimating the effect of the treatment at the quantile of y (log cotinine), conditional on x (e.g. socioeconomic deprivation):

$$CQTE(\tau, \tilde{\boldsymbol{x}}) = Q^{\tau}_{Y(1)|\tilde{\boldsymbol{X}} = \tilde{\boldsymbol{x}},\ \boldsymbol{Z}=0} - Q^{\tau}_{Y(0)|\tilde{\boldsymbol{X}} = \tilde{\boldsymbol{x}},\ \boldsymbol{Z}=0} \#(5)$$

Finally, Inequality Treatment Effect (ITEs)[29] identify the difference in inequality indices between the treated and control:

$$ITE = W(Y(1) \mid \boldsymbol{Z} = 0) - W(Y(0) \mid \boldsymbol{Z} = 0)\#(6)$$

Where $W(.)$ denotes an inequality index, in this case the Concentration Index[30], a *relative* measure of bivariate inequality:

$$W(y,\tilde{x}) = \frac{1}{N}\sum_{i=1}^{N}\left((2R(\tilde{x}_i) - 1)\frac{y_i}{\bar{y}}\right) \#(7)$$

Where $(2R(\tilde{x}_i) - 1)$, is the fractional rank of $\tilde{x}_i$, and $\bar{y} = \frac{1}{N}\sum_{i}^{N}(y_i)$, the mean outcome. The concentration index indicates how concentrated health-related outcomes are amongst those of a higher/lower socioeconomic status. Where there is no socioeconomic-related inequality, the concentration index is zero, with values further from zero indicating greater inequality. We used methods developed by Heckley et al. to estimate ITEs within the regression discontinuity framework using recentered influence function regressions[31].

RESULTS

Baseline Characteristics

Figure 1 shows the distribution of transformed cotinine values (log(cotinine+1)) for non-smokers and smokers in the control group (pre-intervention). For non-smokers, the distribution was heavily right skewed (31.4% of the sample had zero values) with a mean of 0.43 (actual value 4.95 ng/ml). For smokers, the distribution was left skewed with a mean of 5.37 (303.24 ng/ml).

[INSERT FIGURE 1]

Baseline characteristics, by quintile of cotinine, are shown in Appendix A.3. Higher cotinine levels were associated with younger age, being unmarried, living in an urban environment, lower

For peer review only

socioeconomic status, poorer self-assessed health and higher levels of alcohol consumption, in addition to smoking status and tobacco consumption.

Average Treatment Effects

At the time of the ban, the expected baseline level of log cotinine (without treatment) was 0.40 for non-smokers and 5.34 for smokers. There was a large and significant reduction in cotinine concentrations for non-smokers (-0.128, p=0.043), but no significant effect of the ban for smokers (0.036, p=0.817). Regression outputs are shown in Appendix A.4. These results are robust to a set of sensitivity analyses, see Appendix A.5.

These effects are shown at the top of Figure 2, which shows forest plots of average effects of the smoking ban conditional on sex, age, marital status, smoking status of others in the household, equalised household income, highest qualification and IMD (CATEs). For smokers there were no significant effects conditional on any variable. For non-smokers, there were both significant effects and significant differences between subgroups, most notably larger reductions in cotinine levels for younger, single, income poor or IMD deprived respondents.

The differences in CATEs for non-smokers are partially explained by different baseline predictions across subgroups. For example, the predicted average level of log cotinine at the time of the intervention was 0.65 for deprived and 0.30 for non-deprived. After the ban, these levels fell to 0.23 and 0.27 respectively (not significantly different), see Appendix A.6 for more detail.

[INSERT FIGURE 2]

Distributional Analysis

Figure 3 shows Quantile Treatment Effects (QTEs), i.e. the effect of the smoking ban on the level of cotinine at different parts of the distribution (quantiles of cotinine). For non-smokers, there was no effect on cotinine levels at the lower quantiles (which were already at 0) but significant, and increasing,

For peer review only - http://bmjopen.bmj.com/site/about/guidelines.xhtml

reductions appeared at higher quantiles of cotinine. For smokers, there were no significant effects at any quantile.

[INSERT FIGURE 3]

Figure 4 shows QTEs conditional on IMD deprivation. Significant reductions in cotinine levels are apparent for deprived non-smokers at higher levels of exposure. For non-deprived non-smokers there are significant small effects at the mid-range of exposure (going from very little to zero exposure) but not at higher levels. For both groups of smokers there was no significant reduction at any quantile of cotinine.

[INSERT FIGURE 4]

Changes in Inequality

Table 1 shows the causal effect of the ban on bivariate inequality (the concentration index) i.e. how the concentration of log cotinine levels amongst the socioeconomically deprived (IMD quintile) changes.

The negative constants in models (1) and (2) show that higher levels of cotinine were concentrated amongst more socio-economically deprived respondents at baseline for the whole sample (indicating higher levels of smoking) and non-smokers (indicating higher levels of second-hand exposure). However, the constant is close to zero for smokers, therefore conditional on being a smoker, deprivation has limited impact on cotinine levels.

Table 1: Log Cotinine: Concentration Index

	(1) **Sample** Coef./S.E.	(2) **Non-Smokers** Coef./S.E.	(3) **Smokers** Coef./S.E.

For peer review only

Treatment	0.0265	0.2097**	-0.0285
	(0.0693)	(0.0910)	(0.0182)
Time	0.0005	-0.0015*	0.0003**
	(0.0005)	(0.0008)	(0.0001)
Treatment X Time	-0.0009	0.0007	-0.0003
	(0.0008)	(0.0011)	(0.0002)
**Constant**	-0.1501***	-0.2329***	0.0119
	(0.0394)	(0.0559)	(0.0127)
N	3718	2952	766
R-squared	0.0009	0.0029	0.0076

$^{*}p < 0.10$, $^{**}p < 0.05$, $^{***}p < 0.01$

The treatment effect shows that the ban caused a significant increase in the concentration index for non-smokers (i.e. towards zero), reducing the concentration of cotinine levels amongst deprived non-smokers. No significant effects were found for the whole sample or for smokers. These results indicate that the ban caused a reduction in deprivation related inequality in cotinine levels among non-smokers but had no significant effect for smokers.

DISCUSSION

Given the socioeconomic gradient of tobacco smoke exposure, public smoking bans could potentially benefit deprived groups more. Conversely, poorer individuals may respond less well to such policies due to lack of opportunities and resources, contextual factors[32], and attitudes towards anti-smoking policies[33]. We found the smoking ban in England significantly reduced exposure for non-smokers with greater reductions for socioeconomically deprived groups with higher baseline levels of exposure. However, the ban had no significant impact on exposure levels, or on inequalities in exposure, for smokers.

Strengths and limitations of the study

We used a doubly-robust regression discontinuity design to derive causal estimates of the differential impacts of a national smoking ban across the entire distribution of exposure levels, for both smokers and non-smokers. We also used a rich, individual-level dataset with an objective measure of tobacco smoke exposure to avoid social desirability bias in reported variables and explored differential effects

For peer review only

of the ban both between groups using conditional average treatment effects and across the entire distribution of cotinine levels using quantile treatment effects. We unified these approaches to examine conditional distributional effects and estimate the causal impact of the ban on socioeconomic-related inequality in cotinine.

The study had several limitations. First, the HSE is a repeated cross-sectional survey and we were therefore unable to observe individual's over time; rather, we compared distributions of exposure for different individuals surveyed before and after the ban. Second, we relied on self-report to determine smoking status at the time of the survey. The distributions of cotinine levels for smokers and non-smokers suggest that self-report was accurate for the majority of subjects, but there may have been some misclassification. Third, social deprivation was measured by area deprivation rather than by individual social status and was arbitrarily categorised in quintiles, our analysis of socioeconomic deprivation was therefore less fine-grained than our analysis of cotinine levels. Finally, we have only measured the short-term effects of the ban, and these may change over the longer term.

Findings

Our results suggest that the smoking ban in England did little to reduce overall risk or socioeconomic inequalities in smoking related diseases for the smoking population, but it did, as intended, reduce both overall risk and inequalities in risk for non-smokers, with greater reductions in environmental tobacco smoke exposure for socioeconomically deprived non-smokers with higher baseline levels of exposure. These findings align with systematic reviews showing that compulsory smoking restrictions are generally more equitable than voluntary policies[34] and Semple et al.'s findings of greater reductions in environmental smoke in pubs in more deprived areas following the UK ban[21]. A previous study of cotinine levels in non-smokers by Sims et al. similarly reported significant reductions in average exposure following the ban but, in contrast to our results, found no significant reductions in the lowest SES households and a significant reduction for higher SES households (a 37% reduction in the geometric mean of cotinine), implying an increase in bivariate inequality [35]. Whereas our study used a regression discontinuity approach, Sims et al. modelled impacts based on pre-intervention trends that indicated exposure was already falling prior to 2007. However, these models lacked data

For peer review only

for the three years immediately prior to 2007, and may have underestimated additional reductions resulting from the ban. Sims et al. also measured SES using social class of the household head, resulting in a relatively small sample of low status non-smokers; our different results might therefore be explained by reduced levels of exposure being achieved in more deprived areas generally, but not in the very lowest status households. This is consistent with our previous study, which found significant reductions in self-reported exposure for non-smokers which increased with social deprivation, with the exception of individuals with extremely low SES, for whom there was no significant impact[20].

A major concern at the time of the ban was that smokers would substitute smoking in private areas for smoking in public places, and that this could unintentionally increase risk for vulnerable groups, as happened in the US following similar bans[36]. Our results suggest that such substitution activity did not occur and that there was a net reduction in exposure for adult non-smokers; this is supported by findings of reductions in smoking prevalence inside cars (from 32% to 26%) and the home (from 65% to 55%) reported by smokers following the ban[17].

Conclusions

The 2007 smoking ban in England reduced environmental tobacco smoke exposure for non-smokers, with the largest benefits for more deprived non-smokers with high levels of exposure. This group were most at risk from the negative externalities of smoking, and the ban was therefore not only an effective but an equitable intervention. Nevertheless, there might still have been a negative equity impact of the ban across the whole population due to an increased gap in exposure between smokers (who are more likely to be deprived) and non-smokers (who are more likely to be affluent). However, our results show that gaps in overall levels of tobacco smoke exposure narrowed between affluent and deprived non-smokers after the ban and bivariate inequality across the whole population did not increase.

For peer review only

ACKNOWLEDGEMENTS

We would like to thank Richard Cookson, Owen O'Donnell, Elena Ratschen, Kamran Siddiqi and Tom Van Ourti for their helpful comments and enlightening debate.

FUNDING

The study was funded by the Wellcome Trust (205427/Z/16/Z). All errors remain our own.

ETHICAL STATEMENT

This paper performs secondary analysis of publicly available data, therefore, ethical approval was not needed.

COMPETING INTERESTS

The authors have no competing interests to declare.

AUTHOR CONTRIBUTIONS

Authors contributed equally to the paper. JL extracted the data, performed initial analysis and wrote the first draft. MR developed the methods, performed the final analysis and wrote up the methods, results and appendices. TD wrote the abstract, introduction and discussion. All authors critically edited the manuscript.

DATA AVAILABILITY

Data are from the Health Survey for England, 2007 Wave. This is accessible through the UK Data Service, persistent identifier: 10.5255/UKDA-SN-6112-1. This data is safeguarded, and accessible through an end user license.

For peer review only

REFERENCES

[1] Doll, R and Bradford Hill, A. "Smoking and carcinoma of the lung". In: *British Medical Journal* 2.4682 (1950), pp. 739–738.

[2] Wald, N and Nicolaides-Bouman, A. *UK smoking statistics*. Oxford University Press, 1991.

[3] Garfinkel, L. "Time trends in lung cancer mortality among nonsmokers and a note on passive smoking". In: *JNCI: Journal of the National Cancer Institute* 66.6 (1981), 1061–1066.

[4] Hirayama, T. "Non-smoking wives of heavy smokers have a higher risk of lung cancer: a study from Japan." In: *BMJ (Clin Res Ed.)* 282.6259 (1981), 183–185.

[5] Trichopoulos, D. "Lung cancer and passive smoking". In: *International Journal of Cancer* 27.1 (1981), 1–4.

[6] de Waard F, Kemmeren J, van Ginkel L, et al. "Urinary cotinine and lung cancer risk in a female cohort". In: *British Journal of Cancer* 72.3 (1995).

[7] Jee, S, Ohrr, H, and Kim, IS. "Effects of husbands' smoking on the incidence of lung cancer in Korean women." In: *International Journal of Epidemiology* 28.5 (1999), 824–828.

[8] Nishino, Y, Tsubono, Y, Tsuji, I, et al. "Passive smoking at home and cancer risk: a population-based prospective study in Japanese nonsmoking women". In: *Cancer Causes & Control* 12.9 (2001), 797–802.

[9] Fontham, E, Correa, P, Reynolds, P, et al. "Environmental tobacco smoke and lung cancer in nonsmoking women: a multicenter study". In: *Jama* 271.22 (1994), 1752–1759.

[10] Wang, L, Lubin, J, Zhang, S, et al. "Lung cancer and environmental tobacco smoke in a non-industrial area of China". In: *International Journal of Cancer* 88.1 (2000), 139–145.

[11] Hackshaw, A, Law, M, and Wald, N. "The accumulated evidence on lung cancer and environmental tobacco smoke". In: *BMJ* 315.7114 (1997), 980–988.

[12] Health, US Department of, Services, Human et al. *The health consequences of involuntary exposure to tobacco smoke: a report of the Surgeon General*. 2006.

For peer review only

[13] Jaakkola, M, and Samet, J. "Summary: workshop on health risks attributable to ETS exposure in the workplace." In: *Environmental Health Perspectives* 107.suppl 6 (1999), 823–828.

[14] Jamrozik, K. "Estimate of deaths attributable to passive smoking among UK adults: database analysis". In: *Bmj* 330.7495 (2005).

[15] Jha, P, Peto, R, Zatonski, W, et al. "Social inequalities in male mortality, and in male mortality from smoking: indirect estimation from national death rates in England and Wales, Poland, and North America". In: *The Lancet* 368.9533 (2006), 367–370.

[16] Fowkes, F, Marlene, C, Stewart, F, et al. "Scottish smoke-free legislation and trends in smoking cessation". In: *Addiction* 103.11 (2008), 1888–1895.

[17] Lee, J, Glantz, S, and Millett, C. "Effect of smoke-free legislation on adult smoking behaviour in England in the 18 months following implementation". In: *PloS One* 6.6 (2011), e20933.

[18] Jones A, Laporte, A, Rice, N, et al. "Do public smoking bans have an impact on active smoking? Evidence from the UK". In: *Health Economics* 24.2 (2015), 175–192.

[19] England, Health Survey for. *Smoking*. Available at http://healthsurvey.hscic.gov.uk/data-visualisation/data-visualisation/explore-the-trends/ smoking.aspx (2019/09/06).

[20] Robson, M, Doran, T, and Cookson, R. "Estimating and Decomposing Conditional Average Treatment Effects: The Smoking Ban in England". In: *HEDG Working Papers* 19.20 (2019).

[21] Semple, S, Tongeren, M, Galea, K, et al. "UK smoke-free legislation: changes in PM2. 5 concentrations in bars in Scotland, England, and Wales". In: *Annals of occupational hygiene* 54.3 (2010), 272–280.

[22] King, B, Hyland, A, Borland, R , et al. "Socioeconomic variation in the prevalence, introduction, retention, and removal of smoke-free policies among smokers: findings from the International Tobacco Control (ITC) Four Country Survey". In: *International journal of environmental research and public health* 8.2 (2011), 411–434.

[23] Benowitz, N. "Cotinine as a biomarker of environmental tobacco smoke exposure".

For peer review only

In: *Epidemiologic reviews* 18.2 (1996), 188–204.

[24] Jarvis, M, Fidler, J, Mindell, J, et al. “Assessing smoking status in children, adolescents and adults: cotinine cut-points revisited”. In: *Addiction* 103.9 (2008), 1553–1561.

[25] Noble, M, Wilkinson, E, Barnes, H, et al. “The English indices of deprivation 2007”. In: (2008).

[26] Rubin, D. “Estimating causal effects of treatments in randomized and nonrandomized studies.” In: *Journal of Educational Psychology* 66.5 (1974).

[27] Koenker, R, and Bassett G. “Regression quantiles”. In: *Econometrica: Journal of the Econometric Society* (1978), 33–50.

[28] Frandsen, B, Frolich, M,, and Melly, B. “Quantile treatment effects in the regression discontinuity design”. In: *Journal of Econometrics* 168.2 (2012), 382–395.

[29] Firpo, S, and Pinto, C. “Identification and estimation of distributional impacts of interventions using changes in inequality measures”. In: *Journal of Applied Econometrics* 31.3 (2016), 457–486.

[30] Kakwani, N. “Measurement of tax progressivity: an international comparison”. In: *The Economic Journal* 87.345 (1977), 71–80.

[31] Heckley, G, Gerdtham, U, and Kjellsson, G. “A general method for decomposing the causes of socioeconomic inequality in health”. In: *Journal of health economics* 48 (2016), 89–106.

[32] Layte, R and Whelan, C. “Explaining social class inequalities in smoking: the role of education, self-efficacy, and deprivation”. In: *European Sociological Review* 25.4 (2008), 399–410.

[33] Borland, R, Yong, H, Cummings, K, et al. “Determinants and consequences of smoke-free homes: findings from the International Tobacco Control (ITC) Four Country Survey”. In: *Tobacco control* 15.suppl 3 (2006), iii42–iii50.

[34] Brown, T, Platt, S, and Amos, A. “Equity impact of population level interventions and policies to reduce smoking in adults: a systematic review”. In: *Drug and alcohol dependence* 138 (2014), 7–16.

For peer review only

[35] Sims, M. Mindell J, Jarvis M, et al. "Did smokefree legislation in England reduce exposure to secondhand smoke among nonsmoking adults? Cotinine analysis from the Health Survey for England". In: *Environmental health perspectives* 120.3 (2011), pp. 425–430.

[36] Adda, J, and Cornaglia, J. "The effect of bans and taxes on passive smoking". In: *American Economic Journal: Applied Economics* 2.1 (2010), 1–32.

For peer review only

FIGURE LEGENDS

Figure 1

Title: Figure 1: Distribution of Log Cotinine: Non-Smokers and Smokers

Figure 2

Title: Figure 2: Forest Plot of Conditional Average Treatment Effects of Log Cotinine: Non-Smokers and Smokers

Key: Bars denote 95% confidence intervals.

Figure 3

Title: Figure 3: Quantile Treatment Effects: Log Cotinine
Quantile treatment effects for smokers and non-smokers

Key: Solid line denotes Quantile Treatment Effect; dotted line denotes Average Treatment Effect; grey area denotes 90% confidence interval.

Figure 4

Title: Figure 4: Conditional Quantile Treatment Effects: Log Cotinine

Key: Solid line denotes Quantile Treatment Effect; dotted line denotes Average Treatment Effect; grey area denotes 90% confidence interval.

For peer review only

For peer review only

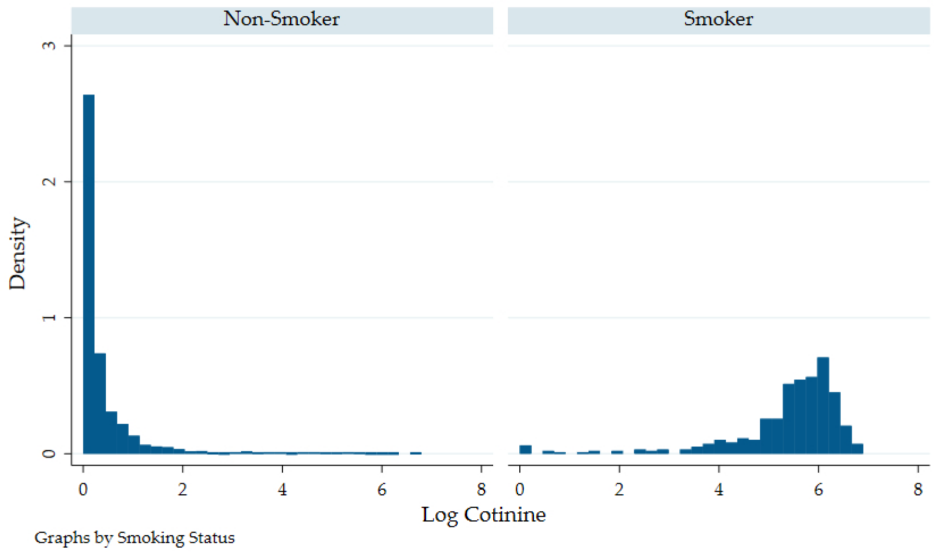

Figure 1: Distribution of Log Cotinine: Non-Smokers and Smokers

310x194mm (72 x 72 DPI)

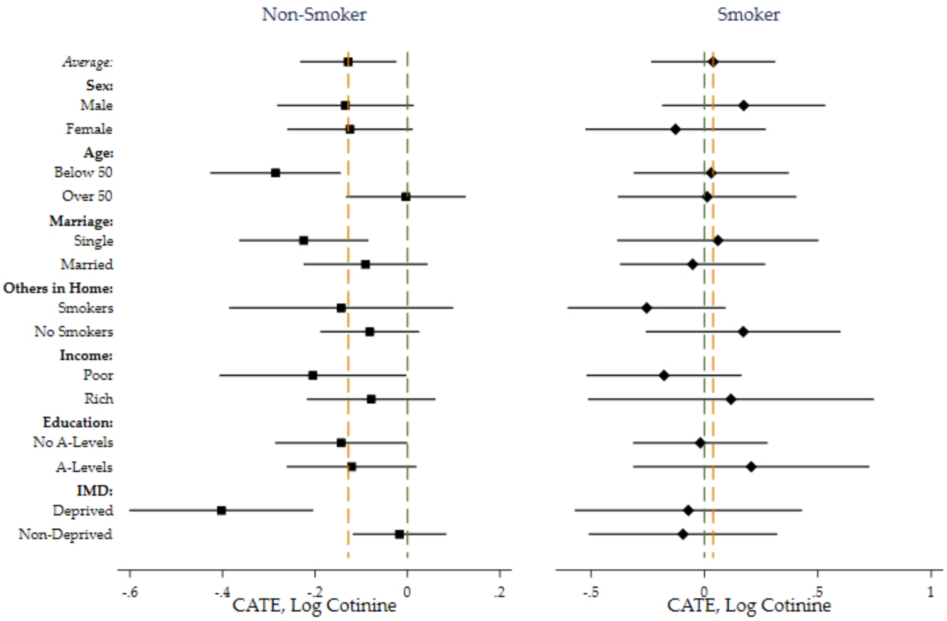

Figure 2: Forest Plot of CATEs of Log Cotinine: Non-Smokers and Smokers

267x194mm (72 x 72 DPI)

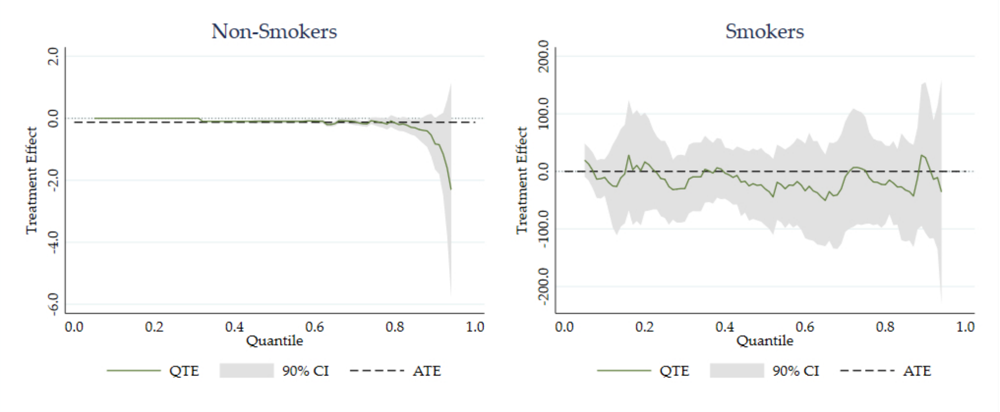

Figure 3: Quantile Treatment Effects: Log Cotinine

358x149mm (72 x 72 DPI)

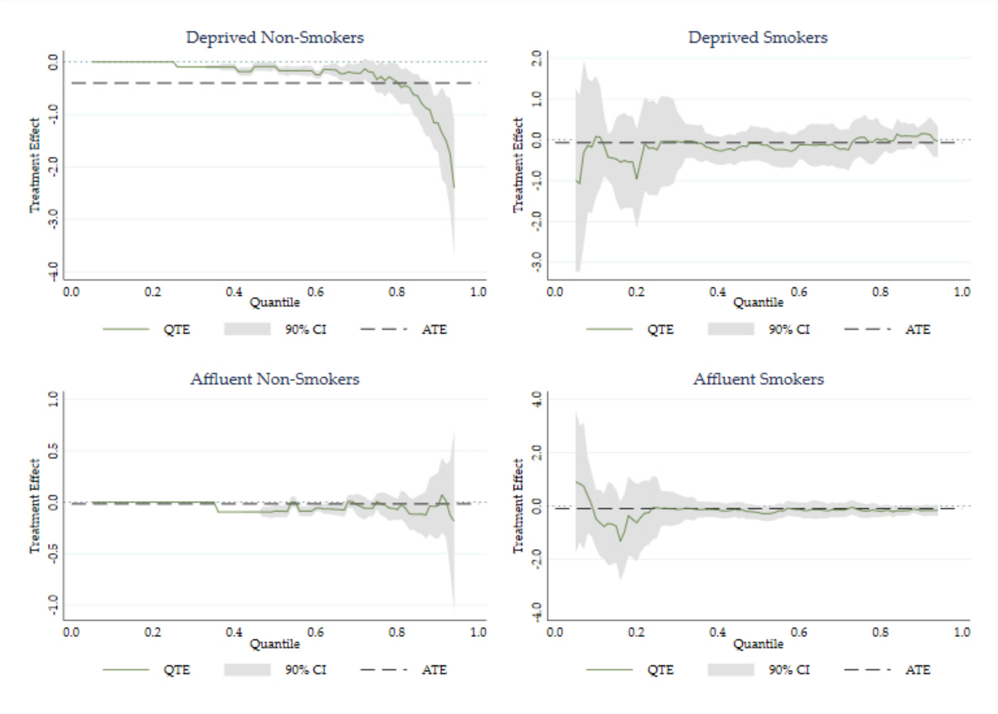

Figure 4: Conditional Quantile Treatment Effects: Log Cotinine

271x194mm (72 x 72 DPI)

A Appendix

A.1 Sensitivity: Cotinine

Throughout the paper we use log(cotinine +1) as our outcome variable. We use this transformed variable due to the highly right skewed nature of cotinine and the potential impact of extreme values on results. Here we present the main results using cotinine, rather than log cotinine. Throughout results are robust to the choice of variable, but standard errors tend to increase for cotinine.

As in the main analysis, for the average treatment effect there are large and significant reductions for non-smokers, and no significant reductions for smokers.

Table A1: Cotinine: Regression Discontinuity

	(1) **Non-Smokers** Coef./S.E.	(2) **Smokers** Coef./S.E.
Treatment	-4.9852**	-12.1932
	(2.5121)	(32.1071)
Time	0.0016	-0.0230
	(0.0293)	(0.2154)
Treatment X Time	0.0501	-0.0729
	(0.0335)	(0.3449)
**Constant**	5.3445**	301.0218***
	(2.4040)	(18.6132)
N	2916	759
R-squared	0.0026	0.0030

* $p < 0.10$, ** $p < 0.05$, *** $p < 0.01$

For conditional average treatment effects, we observe no significant effects for smokers conditional on any variable. For non-smokers, we observe both significant effects and differences between subgroups. The difference between age groups here disappears and standard errors do increase in comparison to the main results. When we estimate these effects using negative binomial regressions, however, these standard errors reduce.

For peer review only

Figure A1: Forest Plot of CATEs of Cotinine: Non-Smokers and Smokers

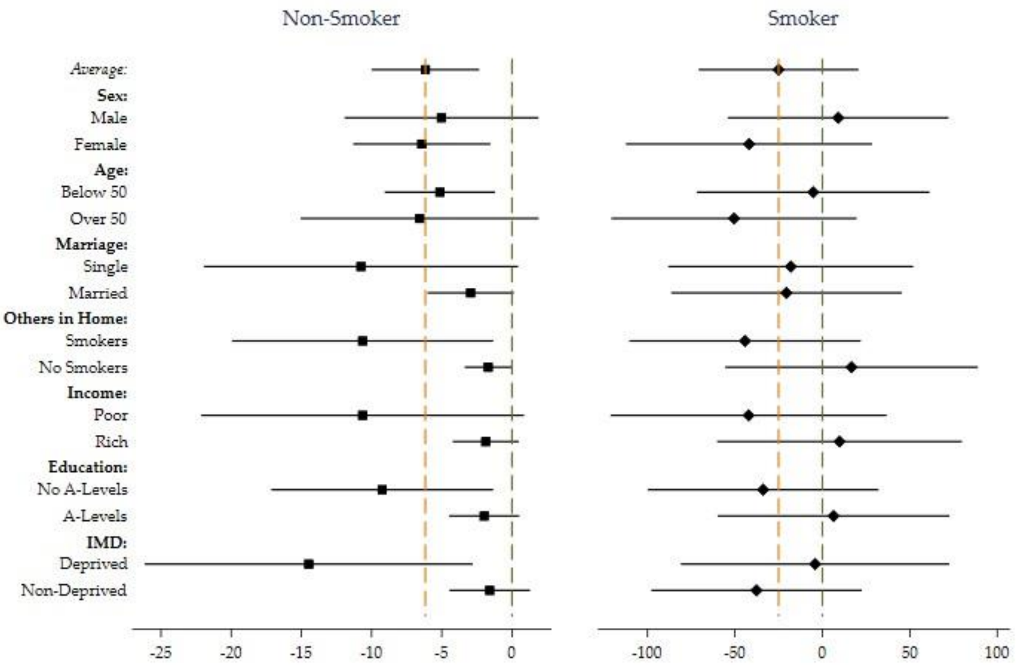

Quantile treatment effects are similar in significance and direction to the main results. There are significant, and increasing, treatment effects for higher quantiles for non-smokers and no significant effects for smokers, at any quantile.

Figure A2: Quantile Treatment Effects: Cotinine

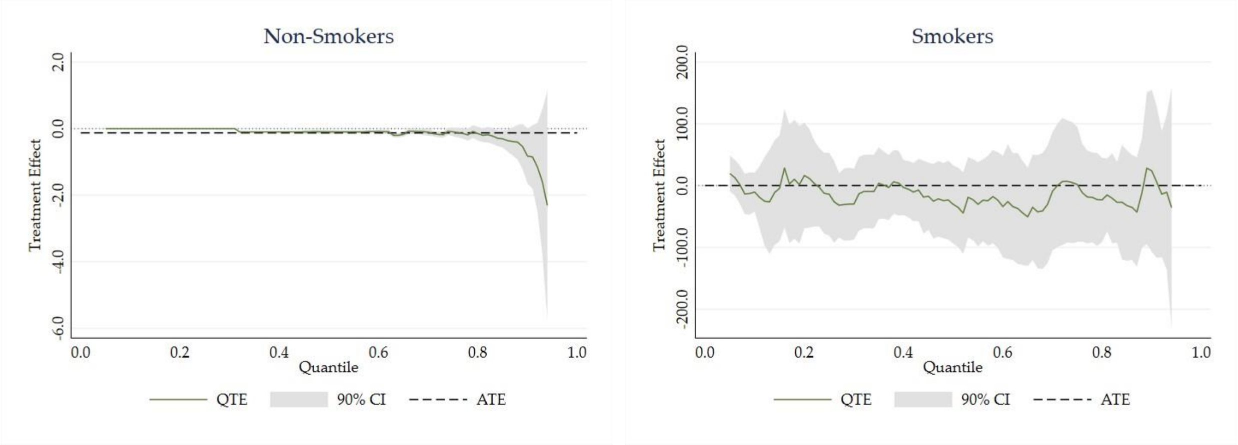

For peer review only - http://bmjopen.bmj.com/site/about/guidelines.xhtml

Inequality treatment effects are very similar to the main results. The concentration of cotinine amongst deprived non-smokers was significantly reduced, whilst no significant effects were observed at the sample level or for smokers.

Table A2: Cotinine: Concentration Index

	(1) Sample Coef./S.E.	(2) Non-Smokers Coef./S.E.	(3) Smokers Coef./S.E.
Treatment	-0.0209	0.5571***	-0.0728
	(0.1031)	(0.1647)	(0.0648)
Time	0.0011	-0.0062**	0.0006
	(0.0008)	(0.0024)	(0.0004)
Treatment X Time	-0.0018	0.0045	-0.0007
	(0.0012)	(0.0031)	(0.0007)
**Constant**	-0.1335**	-0.5714***	0.0356
	(0.0609)	(0.1835)	(0.0379)
N	3675	2916	759
R-squared	0.0010	0.0026	0.0060

* $p < 0.10$, ** $p < 0.05$, *** $p < 0.01$

For peer review only

For peer review only - http://bmjopen.bmj.com/site/about/guidelines.xhtml

A.2 Covariate Balance and Weights

A.2.1 Covariate Balance

Central to the estimation of treatment effects is the comparability of characteristics of the control and treated groups. If there are imbalances in covariates the estimated effects may be biased. To test if there are significant differences in covariates, $x_j,\ \forall j \in J$, the covariate of interest can be regressed against the treatment dummy, *D*:

$$\boldsymbol{x_j} = \beta_0 + \boldsymbol{D}\beta_1 + \boldsymbol{u} \tag{8}$$

Here β_1 identifies the extent and significance of covariate imbalance between control and treated groups. In regression discontinuity designs, however, the crucial assumption is that there are no significant discontinuities in covariates at the *threshold*. Imbalances between control and treated groups *on average* are not important if this assumption holds. In a simple linear framework, this assumption can be tested by regressing the covariates against the treatment dummy, *D*, (centred) forcing variable, *Z* and their interaction:

$$\boldsymbol{x}_j = \gamma_0 + \boldsymbol{D}\gamma_1 + \boldsymbol{Z}\gamma_2 + (\boldsymbol{D}_0 \times \boldsymbol{Z})\gamma_3 + \boldsymbol{u}, \qquad for\ |\boldsymbol{Z}| \leq h \tag{9}$$

If γ_1 is significant it shows there is a discontinuity in $\boldsymbol{x}_j$ at the threshold. This imbalance at the threshold could bias the estimation of the treatment effect.

A.2.2 Inverse Probability of Treatment Weights

To mitigate any potential biases that these imbalances could cause, doubly-robust methods which use propensity weights alongside the regression discontinuity design, can be used. We estimate the inverse probability of treatment to use as weights. This balances covariates across treatment groups, giving lower weight to those characteristics which are predictors of treatment (i.e. via selection into treatment).

For peer review only - http://bmjopen.bmj.com/site/about/guidelines.xhtml

For peer review only

To estimate propensity weights the following regression is run, as a logit:

$$\boldsymbol{D} = \delta_0 + \sum_{j}^{J} \left(\boldsymbol{X_j} \delta_{1j} + \left(\boldsymbol{X_j'} \times |\boldsymbol{Z}| \right) \delta_{2j} \right) + \boldsymbol{u} \qquad for\ |\boldsymbol{Z}| \leq h \tag{10}$$

This formulation includes both covariates $\boldsymbol{X_j}$ and the interaction between those covariates and the absolute value of the forcing variable **Z**. This particular formulation is used to ensure balance not just in expectation (as in Equation (8)) but also at the discontinuity. By using the absolute value of **Z** we provide this flexibility, whilst ensuring the overlap assumption is not violated. These weights are estimated separately for each subgroup.

Using this we predict the probability of being treated, given the observable characteristics $Pr(D = 1|X, Z)$. The inverse probability of treatment weights are then calculated as:

$$w_i^{IPT} = \frac{D}{\Pr(D = 1\,|X, Z)} + \frac{1 - D}{1 - \Pr(D = 1\,|X, Z)} \tag{11}$$

Table A3 shows the consequence of using these weights on covariate balance. Each coefficient (and standard error) are results from separate regressions, with the outcome variable x_j in the first column. Average differences are the β_1 coefficients of Eq.(8), with differences at the discontinuity being the γ_1 coefficients of Eq.(9).

In the unweighted sample we see that the treated group, on average, live in more rural and less deprived areas. At the discontinuity, respondents have significantly fewer children, are more likely to be retired and live in rural areas. These differences could potentially cause bias in the estimates of the treatment effect and need to be controlled for. When the weights are applied, however, we observe no significant differences either on average or at the discontinuity; removing the selection bias (on observables) which could have biased estimates.

For peer review only - http://bmjopen.bmj.com/site/about/guidelines.xhtml

Table A3: Balance

	Unweighted		Weighted	
	(1)	(2)	(3)	(4)
	**Average**	**Discontinuity**	**Average**	**Discontinuity**
	Coef./Std. err.	Coef./Std. err.	Coef./Std. err.	Coef./Std. err.
Age	0.6179	3.5169***	-0.0543	-0.2464
	(0.5288)	(1.0511)	(0.6107)	(1.1586)
Sex (1 = female)	0.0025	-0.0051	0.0005	0.0019
	(0.0149)	(0.0297)	(0.0175)	(0.0337)
Married Couple	-0.0155	-0.0023	-0.0004	0.0026
	(0.0148)	(0.0296)	(0.0173)	(0.0331)
Number of Children	-0.0248	-0.2123***	-0.0007	0.0145
	(0.0239)	(0.0445)	(0.0298)	(0.0592)
Retired	0.0153	0.0829***	-0.0016	-0.0038
	(0.0132)	(0.0266)	(0.0149)	(0.0283)
Ethnicity (1 = white)	0.0170**	0.0089	-0.0023	-0.0056
	(0.0085)	(0.0166)	(0.0106)	(0.0207)
Urban	-0.0088	-0.1027***	0.0034	0.0069
	(0.0126)	(0.0249)	(0.0141)	(0.0254)
IMD Quintile (1 = deprived)	0.0880**	0.1039	-0.0013	-0.0064
	(0.0418)	(0.0838)	(0.0496)	(0.0960)
Occupation: Manager	0.0065	0.0202	-0.0017	-0.0085
	(0.0142)	(0.0284)	(0.0165)	(0.0316)
Education: A-Level	0.0183	-0.0047	-0.0023	-0.0040
	(0.0149)	(0.0297)	(0.0175)	(0.0336)
Home Owner	0.0419***	-0.0038	0.0024	0.0043
	(0.0130)	(0.0259)	(0.0152)	(0.0289)

* $p < 0.10$, ** $p < 0.05$, *** $p < 0.01$

A.2.3 Triangular Kernels

We run each regression discontinuity design as a locally-weighed regression, using triangular kernels to give higher weights to observations closer to the threshold. These weights are calculated as:

$$w_i^{TRI} = h - |Z_i|$$

The highest weights are at the threshold ($Z = 0$) and decrease linearly as the bound of the bandwidth, *h* is reached. These are multiplied by the inverse probability of treatment weights to provide the final weights ($w_i = w_i^{IPT} . w_i^{TRI}$) for the weighted regressions run:

$$\sqrt{w_i}\, \boldsymbol{y} = \sqrt{w_i}\, (\beta_0 + \boldsymbol{D}\beta_1 + \boldsymbol{Z}\beta_2 + (\boldsymbol{D} \times \boldsymbol{Z})\beta_3 + \boldsymbol{u}) \qquad for\ |\boldsymbol{Z}| \leq h \qquad (12)$$

For peer review only - http://bmjopen.bmj.com/site/about/guidelines.xhtml

A.3 Baseline Characteristics

Table A4 summarises the baseline characteristics of the control group, by cotinine quintile. Smoking variables are strongly associated with cotinine, with higher numbers of smokers, higher intensity of smoking and higher exposure to smoke at higher cotinine quintiles. Those with lower levels of cotinine are older, more likely to be female, married and retired, to live in rural areas and have more children. Higher socioeconomic status individuals have lower levels of cotinine, for a range of variables: IMD, income, occupation, education and home ownership. Additionally, those with higher cotinine levels have lower self-assessed health, more limiting longstanding illnesses and they drink more heavily.

Table A4: Descriptive Statistics by Cotinine Quintile

	**Cotinine Quintile**					**Summary**
	**Q1**	**Q2**	**Q3**	**Q4**	**Q5**	**Mean**
	Mean	Mean	Mean	Mean	Mean	Mean
**Cotinine Levels**						
Cotinine	0.00	0.14	0.38	7.61	338.18	69.13
Log Cotinine	0.00	0.13	0.32	1.37	5.68	1.47
**Smoking**						
Smoker	0.01	0.00	0.00	0.12	0.93	0.21
Packs Smoked Per Day	0.00	0.00	0.00	0.14	2.40	0.51
Exposed to Smoke	0.35	0.33	0.44	0.60	0.72	0.48
Hours Exposed to Smoke	1.22	1.18	2.95	7.65	12.37	4.89
Ever Smoked	0.45	0.49	0.54	0.61	0.99	0.61
Smoke in Pubs	0.00	0.00	0.00	0.06	0.28	0.07
Exposed in Pubs	0.16	0.16	0.25	0.38	0.38	0.26
**Demographics**						
Age	52.18	52.35	50.11	48.23	44.80	49.70
Sex (1 = female)	0.54	0.56	0.54	0.52	0.50	0.53
Married Couple	0.67	0.65	0.65	0.54	0.42	0.59
Number of Children	0.46	0.37	0.47	0.40	0.54	0.44
Retired	0.29	0.31	0.28	0.25	0.11	0.25
Ethnicity (1 = white)	0.90	0.91	0.89	0.90	0.92	0.91
Urban	0.76	0.72	0.71	0.80	0.84	0.77
**Socioeconomic Status**						
IMD Quintile (1 = deprived)	3.47	3.44	3.25	2.94	2.62	3.16
Log Equivalised Income	10.14	10.19	10.17	10.01	9.79	10.06
Occupation: Manager	0.42	0.41	0.38	0.31	0.22	0.35
Education: A-Level	0.50	0.49	0.49	0.44	0.33	0.45
Home Owner	0.84	0.78	0.78	0.73	0.54	0.74
**Health Variables**						
Self Assessed Health (5 = v. good)	4.09	4.18	4.08	3.89	3.68	3.99
Limiting Longstanding Illness	0.27	0.23	0.25	0.26	0.33	0.27
Alcohol Units: Heaviest Day	5.34	5.42	6.88	7.81	9.49	6.89

For peer review only - http://bmjopen.bmj.com/site/about/guidelines.xhtml

For peer review only

A.4 Average Treatment Effects

Table A5 shows the results from the regression discontinuity design. The average treatment effect of the ban on non-smokers is large and significant, at -0.128, whilst there is no significant effect for smokers. The constant shows the expected level of log cotinine exposure at the time of the ban, for the control group: 0.399 for non-smokers and 5.343 for smokers.

Table A5: Log Cotinine: Regression Discontinuity

	(1)	(2)
	**Non-Smokers**	**Smokers**
	Coef./S.E.	Coef./S.E.
Treatment	-0.1279**	0.0385
	(0.0632)	(0.1662)
Time	-0.0004	-0.0001
	(0.0005)	(0.0012)
Treatment X Time	0.0010	-0.0006
	(0.0008)	(0.0018)
**Constant**	0.3987***	5.3434***
	(0.0395)	(0.1089)
N	2952	766
R-Squared	0.0062	0.005

$^{*}p < 0.10$, $^{**}p < 0.05$, $^{***}p < 0.01$

Figure A3 shows the regression discontinuity plot for smokers and non-smokers. This shows the (weighted) mean log cotinine (averaged across two days) plotted against the number of days from the ban. The line plot shows the predicted point estimates from the regression of Table A5, whilst the area plot shows the 90% confidence intervals. We observe a discontinuity at the time of the ban for non-smokers, but not smokers. These are the average treatment effects observed in Table A5.

This figure is indicative of the quantile treatment effects we observe in Figure 3: many observations at the lower values of log cotinine move the small distance towards zero, whilst those higher values of log cotinine become much less frequent following the ban.

For peer review only - http://bmjopen.bmj.com/site/about/guidelines.xhtml

Figure A3: Regression Discontinuity Plot: Non-Smokers and Smokers

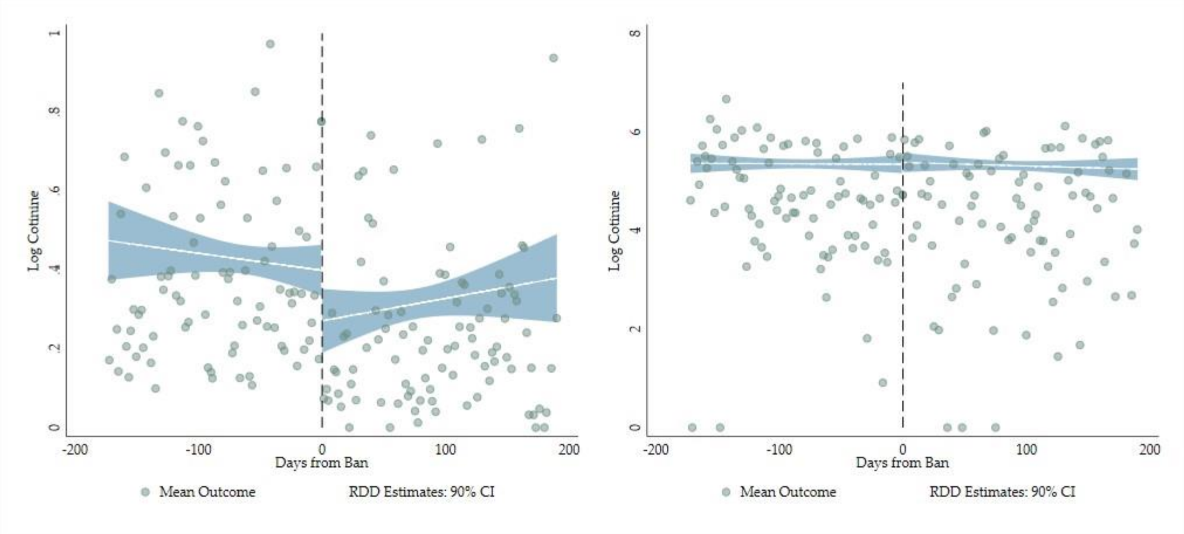

peer review only

A.5 Sensitivity Analysis

Figure A4 shows a forest plot of sensitivity analysis for the estimated average treatment effects. The baseline point estimate of Table 3 is shown at the top, with 90% confidence intervals, for non-smokers and smokers. The estimates below show how sensitive these results are to changes in the functional form of the regression discontinuity, the size of the bandwidth, the weights used and to the addition of controls.

Figure A4: Sensitivity Forest Plot: Log Cotinine, Non-Smokers and Smokers

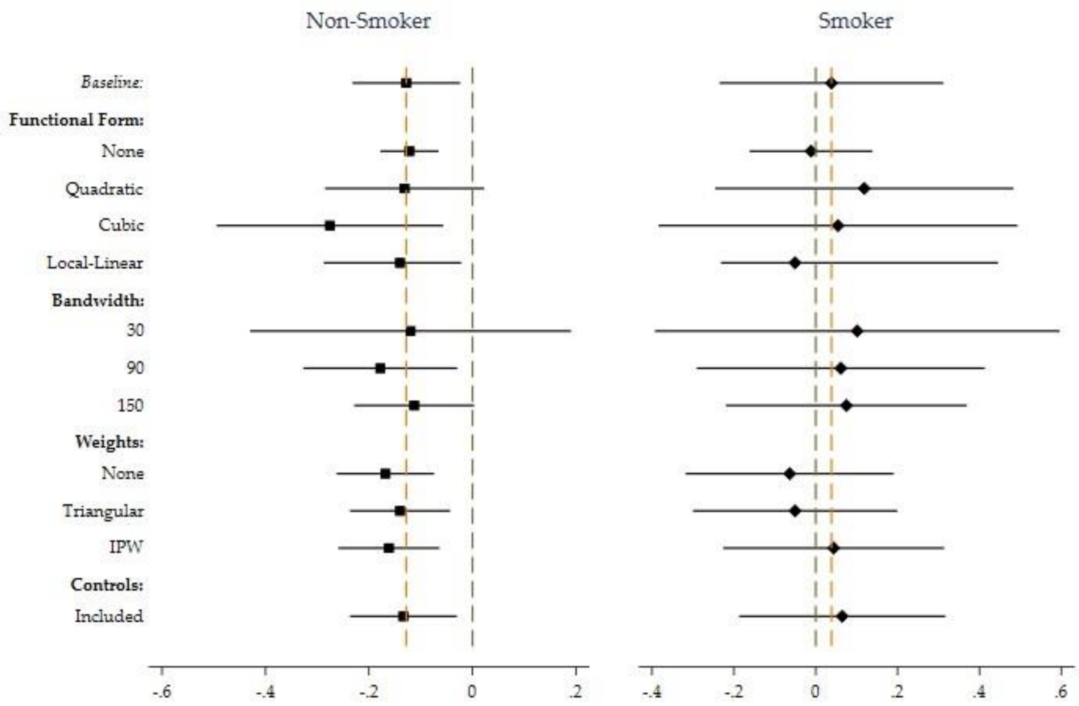

Results show that the analysis appears robust to each of these sensitivity checks. For non-smokers, the effects are never significantly different from the baseline results (dotted orange line) and (with exception to the use of a small bandwidth and quadratic functional form) the estimated effects are always significant, at the 90% level. For smokers, the effects are never significantly different from the baseline effect nor are they significantly different from zero, and indeed appear to hover around a zero effect.

For peer review only - http://bmjopen.bmj.com/site/about/guidelines.xhtml

A.6 Predicted Conditional Potential Outcomes

Figure A5 plots the predicted potential outcomes conditional on observable characteristics. This shows the expected levels of log cotinine at the threshold, for control (red) and treated (blue) groups. These results should be used to supplement the estimated treatment effects in Figure 2; the treatment effects are the differences between these potential outcomes.

Figure A5: Forest Plot of Potential Outcomes of Log Cotinine: Non-Smokers and Smokers

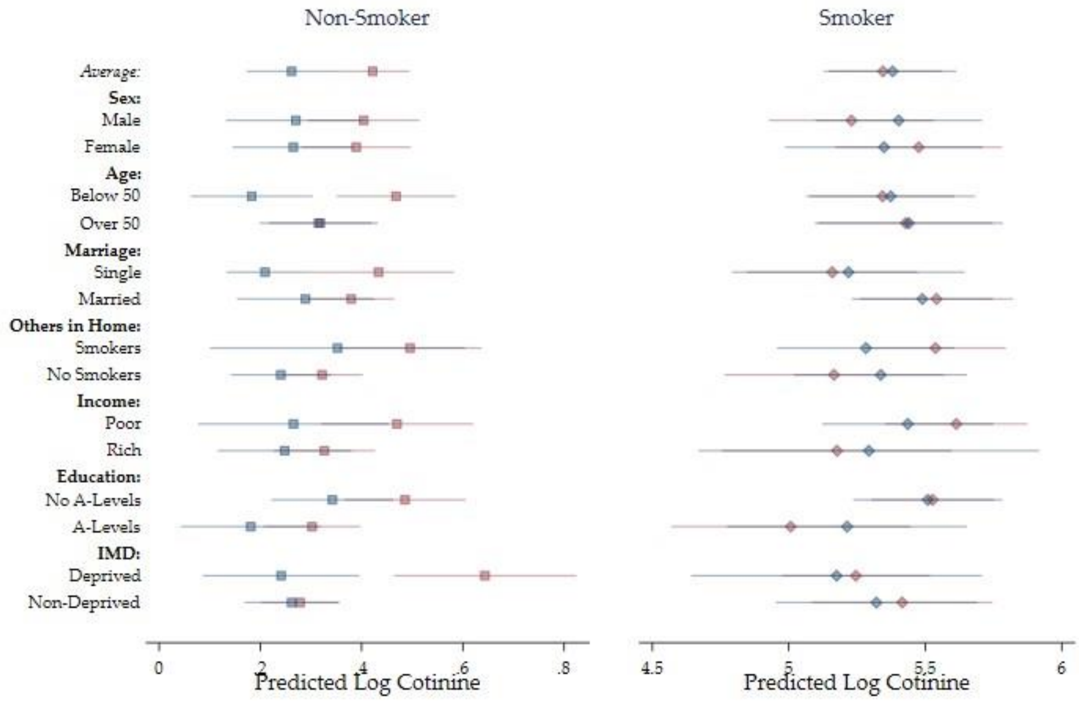

Non-smokers are found to have both significant differences between control and treated potential outcomes (i.e. significant treatment effects) and are found to have differences in exposure levels between subgroups. After the ban the differences between those subgroups that are different before, is much reduced. For smokers, there are no significant differences between potential outcomes; neither between control and treated, nor between subgroups.

For peer review only - http://bmjopen.bmj.com/site/about/guidelines.xhtml
